# ECSIT is a critical limiting factor for cardiac function

**DOI:** 10.1172/jci.insight.142801

**Published:** 2021-06-22

**Authors:** Linan Xu, Fiachra Humphries, Nezira Delagic, Bingwei Wang, Ashling Holland, Kevin S. Edgar, Jose R. Hombrebueno, Donna Beer Stolz, Ewa Oleszycka, Aoife M. Rodgers, Nadezhda Glezeva, Kenneth McDonald, Chris J. Watson, Mark T. Ledwidge, Rebecca J. Ingram, David J. Grieve, Paul N. Moynagh

**Affiliations:** 1 The Kathleen Lonsdale Institute for Human Health Research, Department of Biology, Maynooth University, Maynooth, Ireland.; 2 Wellcome-Wolfson Institute for Experimental Medicine, Queen’s University Belfast, Belfast, United Kingdom.; 3 Center for Biologic Imaging, University of Pittsburgh Medical School, Pittsburgh, Pennsylvania, USA.; 4 Conway Institute, University College Dublin, Dublin, Ireland.; 5 Chronic Cardiovascular Disease Management Unit and Heart Failure Unit, St. Vincent’s Healthcare Group/St. Michael’s Hospital, Dublin, Ireland.

**Keywords:** Cardiology, Metabolism, Cardiovascular disease, Mitochondria

## Abstract

Evolutionarily conserved signaling intermediate in Toll pathways (ECSIT) is a protein with roles in early development, activation of the transcription factor NF-κB, and production of mitochondrial reactive oxygen species (mROS) that facilitates clearance of intracellular bacteria like *Salmonella*. ECSIT is also an important assembly factor for mitochondrial complex I. Unlike the murine form of Ecsit (mEcsit), we demonstrate here that human ECSIT (hECSIT) is highly labile. To explore whether the instability of hECSIT affects functions previously ascribed to its murine counterpart, we created a potentially novel transgenic mouse in which the murine *Ecsit* gene is replaced by the human *ECSIT* gene. The humanized mouse has low levels of hECSIT protein, in keeping with its intrinsic instability. Whereas low-level expression of hECSIT was capable of fully compensating for mEcsit in its roles in early development and activation of the NF-κB pathway, macrophages from humanized mice showed impaired clearance of *Salmonella* that was associated with reduced production of mROS. Notably, severe cardiac hypertrophy was manifested in aging humanized mice, leading to premature death. The cellular and molecular basis of this phenotype was delineated by showing that low levels of human ECSIT protein led to a marked reduction in assembly and activity of mitochondrial complex I with impaired oxidative phosphorylation and reduced production of ATP. Cardiac tissue from humanized *hECSIT* mice also showed reduced mitochondrial fusion and more fission but impaired clearance of fragmented mitochondria. A cardiomyocyte-intrinsic role for Ecsit in mitochondrial function and cardioprotection is also demonstrated. We also show that cardiac fibrosis and damage in humans correlated with low expression of human ECSIT. In summary, our findings identify a role for ECSIT in cardioprotection, while generating a valuable experimental model to study mitochondrial dysfunction and cardiac pathophysiology.

## Graphical abstract

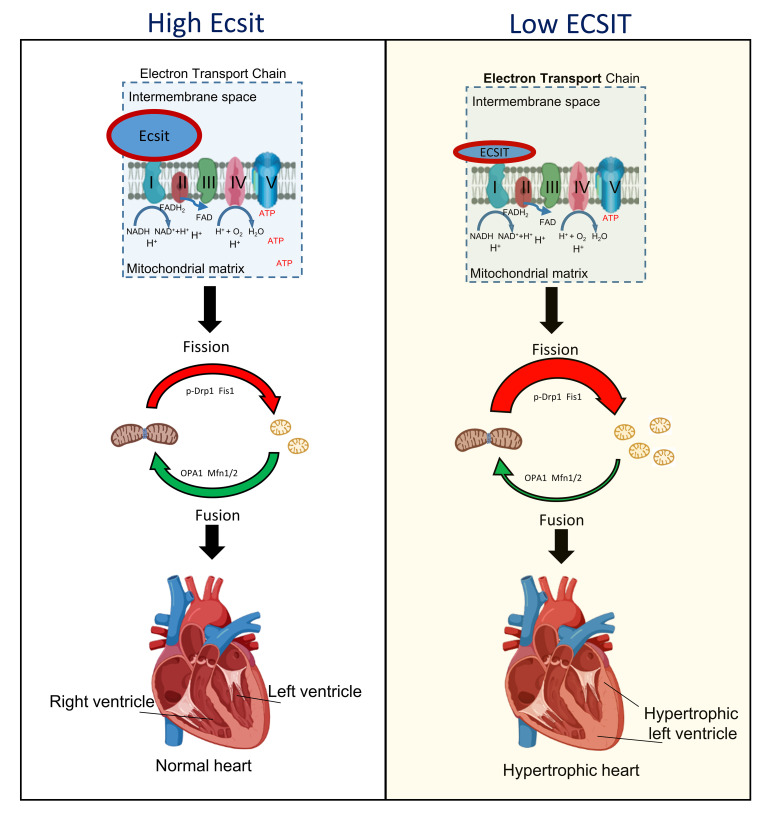

## Introduction

Toll-like receptors (TLRs) recognize pathogen-associated molecular patterns (PAMPs) and respond by inducing the expression of proinflammatory proteins (1). The engagement of TLRs by PAMPs leads to formation of receptor proximal signaling complexes consisting of TIR domain-adaptor proteins, IL-1 receptor–associated kinases (IRAKs), and the E3 ubiquitin ligase TNF receptor–associated factor 6 (TRAF6) (2). Activation of the latter results in its autoubiquitination and formation of unanchored polyubiquitin chains, leading to recruitment of TGF β–activated kinase 1 (Tak1) and downstream activation of the transcription factor NF-κB and MAPK pathways that drive inflammatory gene expression (3). Evolutionarily conserved signaling intermediate in Toll pathways (Ecsit) was first described as a positive regulator of NF-κB by interacting with TRAF6 (4), and more recent reports also indicate its interaction with Tak1 (5) and NF-κB proteins (6). A mutant form of Ecsit that strongly activates NF-κB has been shown to drive hyperinflammatory disease (7). Other studies have identified and characterized Ecsit as part of complex I in the electron transport chain of mitochondria (8–11). An N-terminal mitochondrial localization sequence directs Ecsit to the mitochondria to facilitate assembly of complex I. Furthermore, in macrophages infected with intracellular bacteria such as *Salmonella typhimurium (S*. *typhimurium*), Ecsit can interact with TRAF6 to facilitate the juxtaposition of mitochondria and phagosomes and augment reactive oxygen species (ROS) production to enhance bactericidal activity (12, 13). In macrophages, Ecsit also mediates recruitment of phagosomes to damaged mitochondria to promote removal of the latter by mitophagy (14).

Most analysis of Ecsit function has focused on the murine form of ECSIT (mEcsit) and at the cellular level. Interestingly, across all human tissues, human ECSIT (hECSIT) is expressed at its highest in the heart, but efforts to study the broader physiological role(s) of Ecsit, beyond innate immunity, have been hampered by deletion of the *Ecsit* gene in mice resulting in early embryonic lethality due to impaired bone morphogenetic protein (BMP) signaling during mesoderm formation (15). As part of our efforts to study hECSIT, we noted early in our studies that the hECSIT protein is more labile than its murine counterpart. To understand the physiological relevance of this differential lability, humanized mice were generated in which the *mEcsit* gene was replaced by the *hECSIT* gene. Tissues from humanized hECSIT mice displayed reduced levels of Ecsit, but mice survived and developed normally to adulthood. Macrophages from these humanized mice showed normal activation of the NF-κB and MAP kinase pathways but reduced production of mitochondrial ROS (mROS) and impaired bacterial clearance in response to challenge with *S*. *typhimurium*. Signs of cardiac hypertrophy were apparent at a young age in the humanized *hECSIT* mice and progressed to severe hypertrophy and heart failure in later life. This pathology was associated with marked reduction in subunits and assembly of mitochondrial complex I with impaired oxidative phosphorylation and reduced production of ATP. Cardiac tissue from humanized *hECSIT* mice also showed reduced mitochondrial fusion and more fission but impaired clearance of fragmented mitochondria. The effects of Ecsit depletion on cardiac hypertrophy were also reproduced at a cell level in human cardiomyocytes, supporting a cardiomyocyte-intrinsic role for Ecsit in mitochondrial function and cardioprotection. In human cohorts, we also demonstrate an inverse correlation between levels of hECSIT in heart tissue and cardiac fibrosis and disease. This study adds further understanding to the recognized importance of mitochondrial dysfunction being a major contributor to impaired cardiac functions (16). Pathogenic mutations in core protein subunits and assembly factors of complex I, leading to impaired complex I function, have been reported in a number of diseases, including heart failure and cardiomyopathy (17–19). This study now identifies hECSIT as a critical limiting factor for mitochondrial function and cardioprotection.

## Results

### Human ECSIT is more labile than murine Ecsit.

As part of our efforts to study hECSIT, we initially noted intriguing differences in levels of murine and human forms of ECSIT when we overexpressed myc-tagged forms of both proteins in HEK293T cells (Supplemental Figure 1A, upper panels; supplemental material available online with this article; https://doi.org/10.1172/jci.insight.142801DS1). The steady-state expression levels of hECSIT protein were consistently less than its murine counterpart. Whereas overexpression of mEcsit was sufficient to induce expression of a cotransfected NF-κB–regulated reporter gene, hECSIT was ineffective (Supplemental Figure 1A, lower panels). The inability of hECSIT to activate NF-κB may be due to a potential functional difference from mEcsit or alternatively its low level of expression. Indeed, mEcsit was more resistant to degradation than hECSIT when cells were treated with the protein translation inhibitor cycloheximide, suggesting greater stability for mEcsit (Supplemental Figure 1B). Both proteins migrate as 2 immunoreactive bands with the slower migrating form of hECSIT being especially susceptible to degradation. Both proteins were expressed at comparable levels in extracts from an inducible prokaryotic expression system (Supplemental Figure 1C), but hECSIT was less stable during purification to homogeneity, with greater amounts of purified mEcsit being isolated from similar scales of bacterial cultures (0.75 mg hECSIT versus 1.9 mg mEcsit from 1 L culture, Supplemental Figure 1D). We also compared the relative stabilities of both purified proteins by characterizing resistance to proteolytic processing by the nonspecific protease proteinase K (Supplemental Figure 1E). While both proteins were sensitive to processing, mEcsit was more resistant, suggesting that the folding structure of mEcsit make it more refractory to degradation by proteolysis. The anti-ECSIT antibody showed the same level of immunoreactivity with equal amounts of purified mEcsit and hECSIT (Supplemental Figure 1F), confirming that Western blotting analysis with this antibody can differentiate between varying levels of mEcsit and hECSIT and excluding the possibility of differential immunoreactivity of the 2 forms of protein with the antibody.

Given the more labile nature of hECSIT coupled to its inability to activate NF-κB, we were keen to explore the physiological relevance, if any, of these differences with mEcsit. We thus created a humanized mouse in which the mouse *Ecsit* gene was replaced by the human *ECSIT* gene under the control of the endogenous murine *Ecsit* promoter. Homologous recombination was used in embryonic stem cells to replace the murine coding exons 3–9 with the human coding exons 2–8 (Supplemental Figure 2A), as confirmed by PCR analysis (Supplemental Figure 2B). Heterozygous knockin mice (*ECSIT*^+/–^) containing a copy each of the murine *Ecsit* and human *ECSIT* alleles were viable, and these mice were inbred to generate homozygous knockin mice (*ECSIT*^+/+^) containing 2 copies of the human *ECSIT* allele. The crosses of heterozygous mice resulted in WT, heterozygous, and homozygous mice with frequencies predicted by Mendelian genetics (Supplemental Figure 1C). The homozygous human *ECSIT*–knockin mice are viable and develop normally in early age. All studies used littermate mice derived from crossing of heterozygous human *ECSIT*–knockin mice. Since previous studies have shown that knockout of the mouse *Ecsit* gene results in early developmental lethality due to loss of BMP signaling (15), the survival and development of homozygous human knockin mice containing the human ECSIT alleles clearly indicate that the human ECSIT protein can functionally substitute for the murine equivalent in this early developmental pathway. We next examined if hECSIT could similarly fulfill the other previously described roles for mEcsit in activation of the NF-κB pathway and clearance of intracellular bacteria.

### Bone marrow–derived macrophages from hECSIT-knockin mice show normal activation of NF-κB and proinflammatory cytokine expression but impaired mROS production and bacterial clearance.

**Figure 1:**
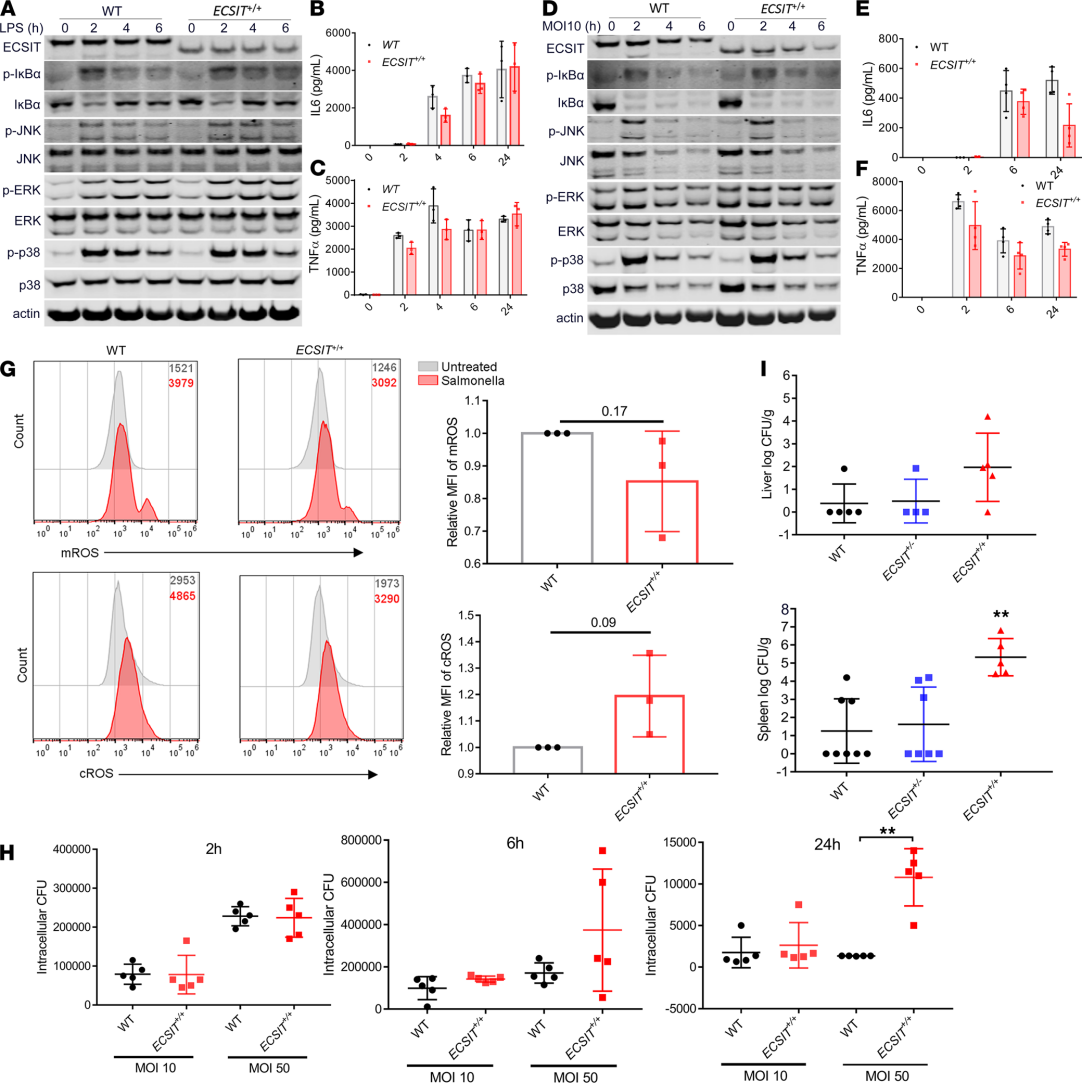
Humanized ECSIT mice show intact activation of NF-κB and MAPK pathways but impaired production of mROS and clearance of *S.* *typhimurium*. (**A**) Immunoblot analysis of ECSIT, β-actin, and total and phosphorylated (p-) forms of IκBα, JNK, ERK, and p38 MAPK in cell lysates isolated from WT and *ECSIT^+/+^* BMDMs after LPS stimulation (100 ng/mL) for the indicated time periods. (**B** and **C**) ELISA analysis of (**B**) IL-6 and (**C**) TNF-α in cell supernatants of WT and *ECSIT^+/+^* BMDMs after LPS stimulation (100 ng/mL) for 0 to 24 hours. (**D**) Immunoblot analysis of ECSIT, β-actin, and total and phosphorylated (p) forms of IκBα, JNK, ERK, and p38 MAPK in cell lysates isolated from WT and *ECSIT^+/+^* BMDMs after challenge with heat-inactivated *S*. *typhimurium* (strain SL1344) for indicated times at an estimated multiplicity of infection (MOI) of 10. (**E** and **F**) ELISA analysis of (**E**) IL-6 and (**F**) TNF-α in cell supernatants of WT and *ECSIT^+/+^* BMDMs after infection. (**G**) MitoSox (mROS) and CM-H2DCFDA (cROS) staining of WT and *ECSIT^+/+^* BMDMs infected *S*. *typhimurium* with MFI expressed relative to uninfected WT cells. (**H**) CFU analysis of BMDMs from WT and *ECSIT^+/+^* mice after 2- to 24-hour infection with *S*. *typhimurium* SL1344 at MOI of 10 and 50. (**I**) CFU analysis of homogenized liver and spleen tissue from WT, *Ecsit*^+/–^, and *ECSIT^+/+^* mice after infection for 72 hours by oral gavage with *S*. *typhimurium* SL1344 (1 × 10^7^ CFU). Data are expressed as the mean ± SD from at least 3 technical replicates, and each representation has at least 2 independent experiments (**A**–**H**); unpaired 2-tailed Student’s *t* test, **P < 0.01. Data indicate samples from individual mice (*n* = 5–8) (**I**); 1-way ANOVA with Dunnett’s multiple comparisons test, **P < 0.01.

Bone marrow–derived macrophages (BMDMs) were prepared from *ECSIT*^+/+^ mice, and again cellular protein levels of hECSIT were lower than mEcsit from WT mice (Figure 1A). However, *ECSIT*^+/+^ BMDMs showed similar responsiveness as WT cells to LPS in terms of temporal activation of the NF-κB and MAPK pathways (Figure 1A) and induction of the proinflammatory cytokines IL-6 (Figure 1B) and TNF-α (Figure 1C). Furthermore, infection of *ECSIT*^+/+^ and WT BMDMs with *S*. *typhimurium* showed comparable activation of NF-κB and MAPK pathways (Figure 1D) and induction of IL-6 (Figure 1E) and TNF-α (Figure 1F). These findings suggest that lower levels of hECSIT do not limit the early signaling pathways and proinflammatory cytokine expression triggered by LPS and TLR4. The inability of overexpressed hECSIT to activate NF-κB in 293T cells (Supplemental Figure 1A) suggests a cell type–specific role for hECSIT in this pathway, or alternatively ECSIT is required but not sufficient for activation of NF-κB and may require other cellular components to be triggered by LPS and the TLR4 pathway. However, BMDMs from *ECSIT*^+/+^ mice displayed a tendency toward reduced mROS but not cytosolic ROS (cROS), relative to WT cells, in response to infection by *S*. *typhimurium* (Figure 1G). This suggests that the lower levels of hECSIT are critical to the mitochondrial function of Ecsit at least in the context of mROS production. Given the importance of Ecsit in promoting mROS production to effect bactericidal activity against *S*. *typhimurium*, we also investigated the capacity of cells from *ECSIT*^+/+^ mice to clear *S*. *typhimurium*. At higher MOI, BMDMs from *ECSIT*^+/+^ mice displayed increased bacterial burden, relative to WT BMDMs, at 24 hours postinfection by *S*. *typhimurium* (Figure 1H). The physiological relevance of this limiting aspect was confirmed in an in vivo infection model in which *ECSIT*^+/+^ mice, orally infected with *Salmonella*, demonstrated impaired clearance and increased dissemination to the liver and spleen when compared with WT and *ECSIT*^+/–^ mice (Figure 1I).

### Human ECSIT–knockin mice develop early cardiac hypertrophy leading to premature death.

**Figure 2:**
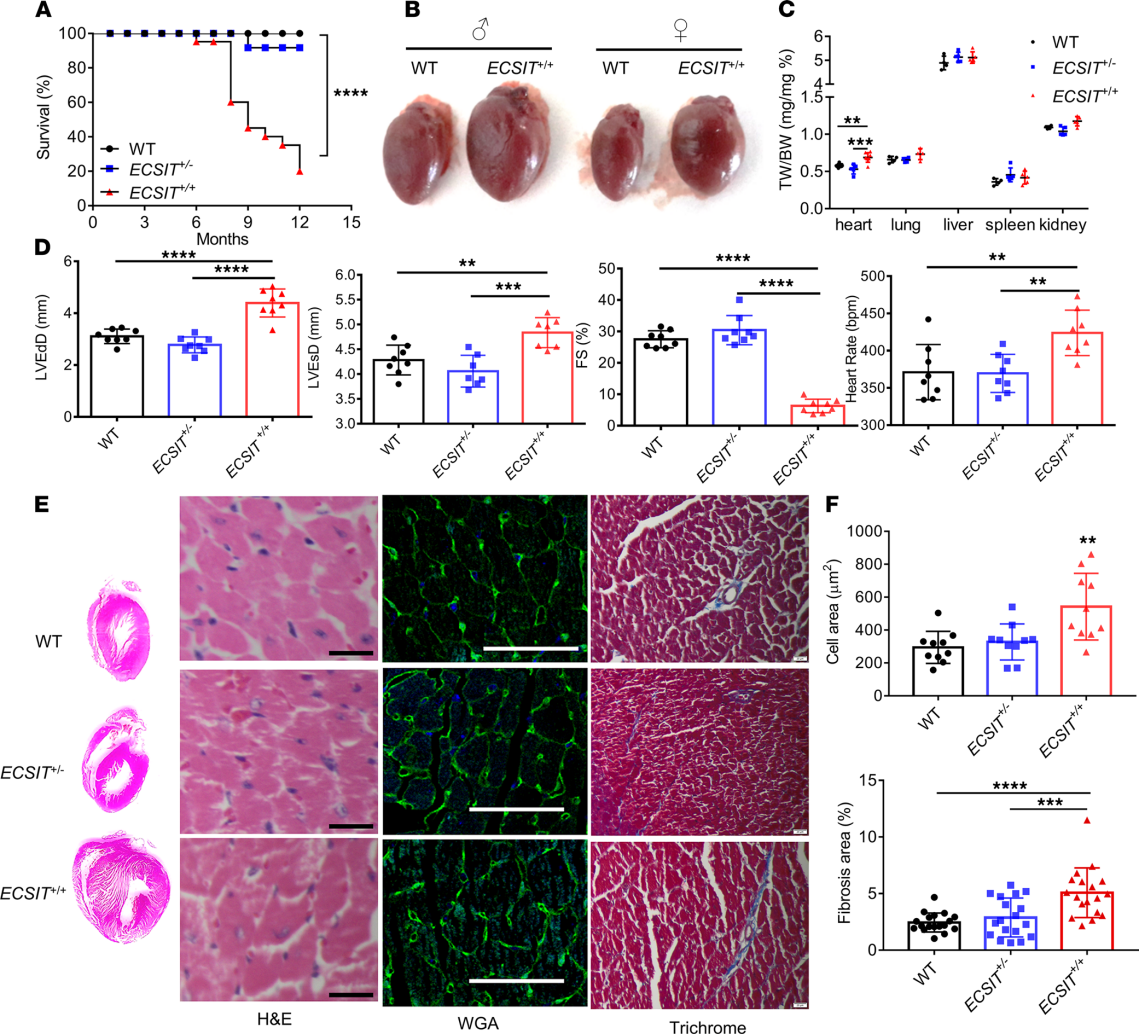
Humanized ECSIT mice develop cardiac hypertrophy leading to premature death. (**A**) Survival rates from a cohort of age- and sex-matched WT, heterozygous (*ECSIT^+/–^*), and homozygous (*ECSIT^+/+^*) mice (*n* = 20) over a 12-month time period. ****P < 0.0001 represents a Mantel-Cox log-rank test of survival analysis. (**B**) Representative images of hearts from male and female WT and *ECSIT^+/+^* mice at 7 months of age. (**C**) Ratios of tissue weight/body weight (TW/BW) of WT and *ECSIT^+/+^* mice (*n* = 4–8) at 8–10 weeks. (**D**) Echocardiograph scans of WT, *ECSIT^+/–^*, and *ECSIT^+/+^* mice (*n* = 8) at 6–7 months of age measuring left ventricular end-diastolic diameter (LVEdD), left ventricular end-systolic diameter (LVEsD), fractional shortening (FS), and heart rate. (**E**) Representative H&E, WGA, and trichrome staining of left ventricular heart tissue from 6–7 months WT, *ECSIT^+/–^*, and *ECSIT^+/+^* mice. (**F**) Quantification of WGA determined cell area and trichrome-based fibrosis scores; *n* = 10 and 18, respectively. (**C**, **D**, and **F**) Data are expressed as the mean ± SD; 1-way ANOVA with Dunnett’s multiple comparisons test, **P < 0.01, ***P < 0.001, ****P < 0.0001. Scale bar: (**E**) 20 μm (H&E); 50 μm (WGA); 20 μm (trichrome).

While *ECSIT*^+/+^ mice showed normal development and maturation into adulthood, we noted that as *ECSIT*^+/+^ mice aged, they were subject to premature death from heart failure, with some mice dying at 8 months of age and less than 20% of the mice being viable at 12 months (Figure 2A). The viability of *ECSIT*^+/–^ mice over this period was comparable with WT mice. Postmortem analysis displayed very distinct enlargement of hearts in *ECSIT*^+/+^ mice relative to age- and sex-matched WT mice (Figure 2B). The greater heart/body weight ratio was evident from 8–10 weeks of age and further increased at 7 months (Figure 2C and Supplemental Figure 3A). Tissue enlargement in *ECSIT*^+/+^ mice was restricted to the heart since other tissues, such as lung, liver, spleen, and kidney showed the same size and weight in WT and *ECSIT*^+/+^ mice (Figure 2C and Supplemental Figure 3A). Notably, heart enlargement was only apparent in homozygous *ECSIT*^+/+^ and not heterozygous *ECSIT*^+/–^ mice (Figure 2C and Supplemental Figure 3A). Echocardiographic analysis was performed on 6- to 7-month-old mice in order to further explore and define the effects of knockin of human *ECSIT* on cardiac physiology. Echocardiography confirmed marked cardiac hypertrophy (increased left ventricular posterior wall systole [LVPWS], left ventricular posterior wall diastole [LVPWD]; Supplemental Table 2) together with significant chamber dilatation (increased left ventricular end-diastolic diameter [LVEdD]; Figure 2D) and systolic (increased left ventricular end-systolic diameter [LVEsD], decreased fractional shortening [FS]; Figure 2D) and diastolic dysfunction (increased isovolumetric relaxation time [IVRT] and myocardial perfusion imaging [MPI]; Supplemental Table 2) in *ECSIT*^+/+^ but not WT or heterozygous hearts, despite compensatory increases in heart rate observed in *ECSIT*^+/+^ mice (Figure 2D). Notably, these major structural and functional alterations were associated with significant echocardiogram changes, specifically prolonged P-R interval and QRS complex duration (Supplemental Table 2), suggestive of electrophysiological dysfunction. Histological analysis of cardiac tissue using H&E staining highlighted abnormal tissue architecture in humanized hearts (Figure 2E). Wheat germ agglutinin (WGA) staining of cell membrane proteins (Figure 2E) allowed for calculation of cardiomyocyte cross-sectional area (Figure 2F) and confirmed cardiac hypertrophy at the cellular level, in cardiac tissue from *ECSIT*^+/+^ mice. Heart tissue from *ECSIT*^+/+^ also displayed clear signs of damage and fibrosis as indicated by greater trichrome staining of collagen volume (Figure 2, E and F). *ECSIT*^+/+^ heart tissue also showed more intense immunostaining of extracellular matrix proteins laminin and collagen VI and the fibrotic marker periostin (Supplemental Figure 3B). In an effort to understand if cell apoptosis underpinned the cell damage, we performed TUNEL staining (Supplemental Figure 4, A and B) and analyzed levels of cleaved caspase-3 (Supplemental Figure 4C). However, TUNEL staining and caspase-3 cleavage were comparable in heart tissue from WT and *ECSIT*^+/+^ mice, suggesting that necrosis rather than apoptosis is the likely mechanism of any cardiomyocyte cell death in *ECSIT*^+/+^ mice.

Given our earlier findings of reduced stability of hECSIT relative to mEcsit, we assessed the expression levels of the murine and human forms of these proteins in heart tissue from WT and *ECSIT*^+/+^ mice, respectively. Western blot analysis clearly demonstrated greatly reduced levels of hECSIT in *ECSIT*^+/+^ heart relative to levels of mEcsit in WT heart with *ECSIT*^+/–^ heart displaying expression of both forms (Supplemental Figure 3C, *lower panel*). Interestingly, quantitative reverse transcriptase PCR (RT-PCR) analysis demonstrated comparable levels of mRNAs encoding mEcsit and hECSIT in cardiac tissue from WT, *ECSIT*^+/–^, and *ECSIT*^+/+^ mice, consistent with our proposed model of hECSIT protein being less stable than mEcsit (Supplemental Figure 3C, *upper panel*). The decreased levels of hECSIT relative to mEcsit was apparent in hearts from male and female mice (Supplemental Figure 3D). The relatively lower levels of hECSIT protein relative to mEcsit was also apparent in other tissues from male and female mice, including spleen, lung, liver, and kidney, although the biggest difference in levels was observed in the heart (Supplemental Figure 3E). All of these data are consistent with hECSIT being less stable than mEcsit with the reduced steady-state levels of hECSIT underlying cardiac hypertrophy and fibrosis and threshold levels being required for normal cardiac function.

### Low levels of human ECSIT correlate with cardiac hypertrophy and fibrosis.

**Figure 3:**
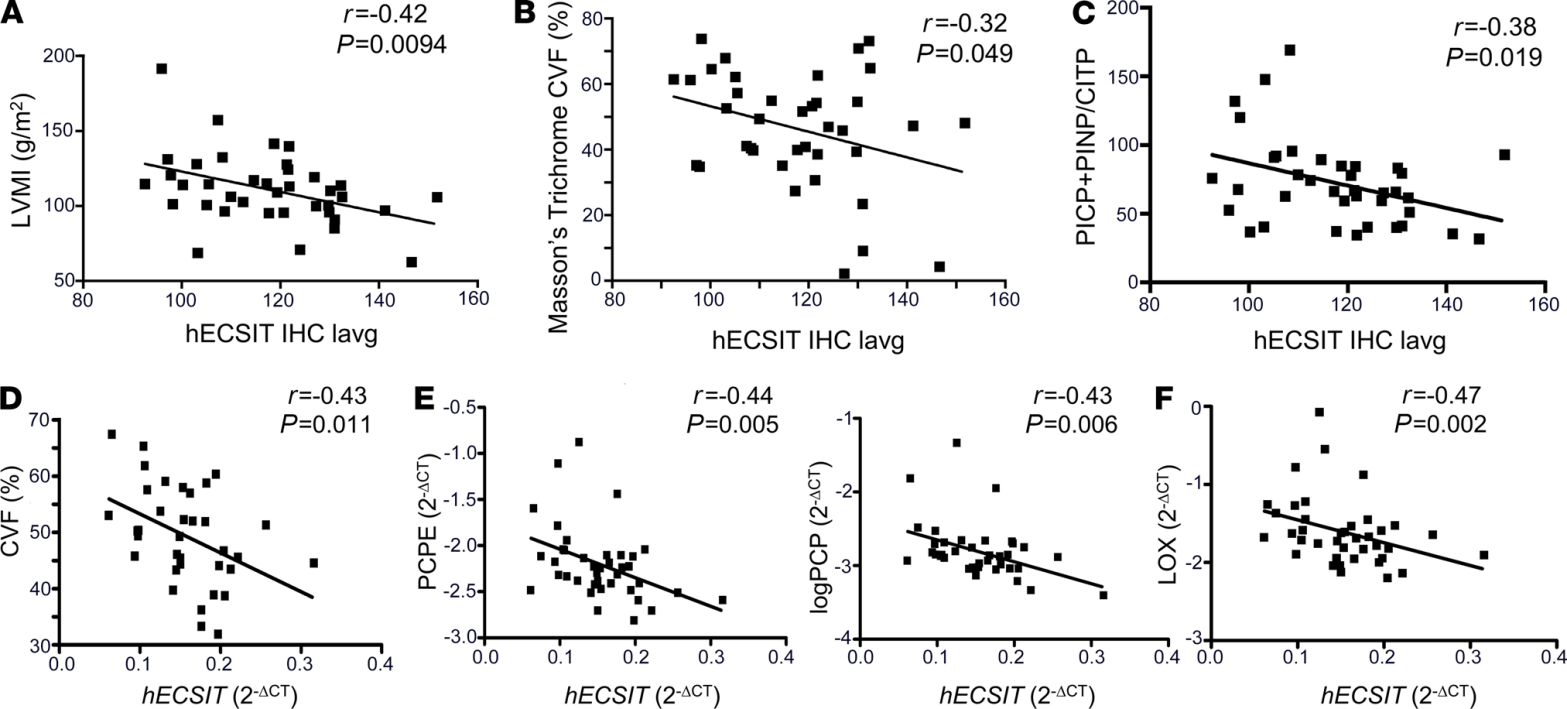
ECSIT negatively correlates with human left ventricular hypertrophy and cardiac fibrosis. (**A**) LVMI. (**B**) Masson’s trichome collagen volume fraction. (**C**) Negative correlations between ECSIT IHC average intensity of all pixels (lavg) and markers of collagen turnover; PICP+PINP/CITP ratio *r* = –0.38, *P* = 0.019. (**D**) Negative correlations between ECSIT mRNA and collagen volume fraction (CVF). (**E**) Negative correlations between ECSIT mRNA and human cardiac PCPE mRNA and PCP mRNA. (**F**) Negative correlations between ECSIT mRNA and human cardiac lysyl oxidase (LOX) mRNA. The study utilized a cohort of 38 patients. The relationships between ECSIT and markers of collagen turnover were assessed using Pearson’s or Spearman’s rank order correlations for variables that were normally or non-normally distributed, respectively.

Given that low levels of hECSIT manifest in cardiac hypertrophy, we are keen to examine if this relationship had pathophysiological relevance in humans. Intriguingly, gene expression analysis across 86 human tissues/cell types showed hECSIT to be expressed at its highest level in the heart (Supplemental Figure 5; BioGPS, http://biogps.org/#goto=genereport&id=51295; refs. 20–23). We next examined ex vivo human cardiac tissue samples from patients undergoing cardiac surgery and explored the association of hECSIT gene and protein expression in the context of human cardiac hypertrophy and fibrosis. Briefly, cardiac tissue samples were obtained from the right atrial appendage adjacent to the venous cannulation site in 38 consecutive, consenting patients undergoing elective surgery for coronary artery bypass grafting or valve replacement. In addition, serum samples for biomarker analyses and Doppler echocardiographic images were obtained. Patients were aged 68.0 ± 9.6 years, 28 (74%) were male, 10 (26%) were female, 27 (71%) had ischemic heart disease, 19 (47%) had valvular heart disease, and 16 (42%) had left ventricular hypertrophy (>95 g/m^2^ for women and >115 g/m^2^ for men) (24). Patients did not have evidence of overt heart failure, and most patients had a normal ejection fraction (>50%). Other patient characteristics and clinical demographic and Doppler Echocardiographic criteria are presented in Supplemental Table 1, according to the presence or absence of evidence of left ventricular hypertrophy. We performed immunohistochemical analysis and quantitative RT-PCR on cardiac tissue samples from these cohorts in order to measure protein and mRNA levels of hECSIT. While immunohistochemistry indicated that there was no association between levels of hECSIT protein and a number of echocardiographic parameters, there was a significant negative correlation between hECSIT protein expression in the heart and left ventricular mass index (LVMI; Figure 3A), suggesting that low cardiac hECSIT protein expression is associated with left ventricular hypertrophy. Furthermore, hECSIT protein also demonstrated negative correlation with indicators of cardiac damage and fibrosis, such as Masson’s trichrome staining for collagen volume (Figure 3B and Supplemental Figure 6). In addition, cardiac levels of hECSIT protein were inversely related to serum markers of collagen I turnover denoted by the ratio of carboxy- and aminoterminal propeptides of procollagen (PICP and PINP, markers of collagen biosynthesis) to carboxyterminal telopeptide of type I collagen (CITP; marker of collagen breakdown; Figure 3C). Collagen I is the predominant form of collagen in the human heart, and these data are consistent with low levels of cardiac hECSIT expression being associated with accumulation of collagen and cardiac fibrosis. We also explored association of cardiac levels of mRNA encoding *hECSIT* and indicators of fibrosis. *hECSIT* mRNA was negatively associated with collagen volume as revealed by Picrosirius red staining (Figure 3D). This is also consistent with *hECSIT* mRNA inversely correlating with mRNA levels of genes encoding the procollagen processing enzymes procollagen C-proteinase enhancer (PCPE) and procollagen C-proteinase (PCP; Figure 3E) and lysyl oxidase that catalyzes cross-linking of collagen molecules (Figure 3F). These data are consistent with high-level expression of ECSIT being important for cardiac function and protection, with low-level expression being associated with cardiac hypertrophy and fibrosis.

### Heart from human ECSIT–knockin mice is characterized by reduced levels of mitochondrial complex I proteins.

**Figure 4:**
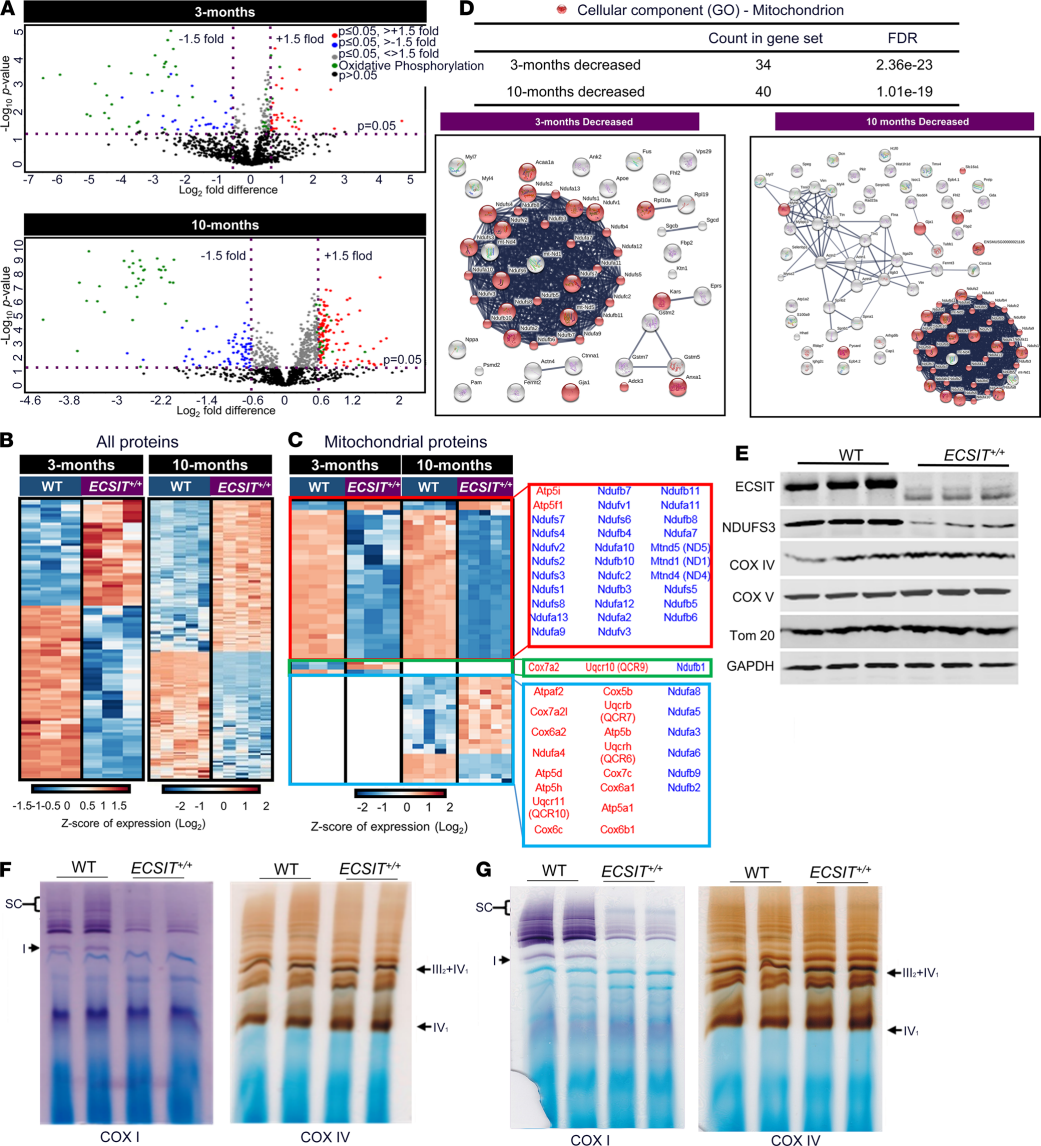
Proteomic analysis of humanized hECSIT heart. (**A**) Volcano plot analysis of differentially expressed proteins in heart tissue from 3- and 10-month-old WT and *ECSIT^+/+^* mice. (**B**) Heatmap analysis of differentially expressed proteins in heart tissue from 3- and 10-month-old WT versus *ECSIT^+/+^* mice. (**C**) Heatmap analysis of differentially expressed oxidative phosphorylation-associated mitochondrial proteins in heart tissue from 3- and 10-month-old WT versus *ECSIT^+/+^* mice. (**D**) STRING network analysis of statistically significant differentially expressed proteins of decreased abundance in heart tissue from 3- and 10-month-old WT and *ECSIT^+/+^* mice. Mitochondrial proteins are highlighted in red. FDRs are computed *P* values corrected for multiple testing using the method of Benjamini and Hochberg. Data indicate samples from individual mice (*n* = 3–5 for all groups). (**E**) Immunoblot analysis of left ventricular cardiac tissue for levels of ECSIT, NDUFS3, Cox IV, Cox V, translocase of outer membrane 20 (Tom20), and GAPDH in WT and *ECSIT^+/+^* mice (*n* = 3) of 8 to 10 weeks of age. (**F** and **G**) Isolated heart mitochondria from WT and *ECSIT^+/+^* mice at (**F**) 7 months (*n* = 2) and (**G**) 10 weeks of age (*n* = 2) were subjected to BN-PAGE followed by in-gel activity assays using complex I– and complex IV–specific substrates. Complex I activity, as an individual complex or as part of supercomplexes (SC), is shown in violet. Complex IV activity, as an individual complex or as part of supercomplex with complex III, is shown in brown.

We next assessed the molecular basis of the cardiac hypertrophy and dysfunction that are associated with low expression of hECSIT. To this end, we utilized an unbiased proteomic approach to systematically and quantitatively analyze dynamic differences between WT and *ECSIT*^+/+^ in their heart proteomes at 3 months and 10 months of age. Using liquid chromatography mass spectrometry label-free quantitation and bioinformatics analysis, 1177 and 1334 proteins were robustly identified in WT versus *ECSIT^+/+^* hearts at 3 months and 10 months, respectively (Figure 4A and Supplemental Figure 7, A and B). Restriction of differential expression to at least 2 unique peptide matches and fold changes of at least 1.5 revealed altered expression of 95 proteins when comparing WT with *ECSIT*^+/+^ mouse hearts at 3 months of age, with 36 proteins showing increased abundance and 59 proteins exhibiting decreased abundance in the *ECSIT*^+/+^ hearts (Figure 4B and Supplemental Table 3). At 10 months of age, 228 proteins exhibited a significant differential expression pattern, with 124 being increased and 104 decreased in *ECSIT*^+/+^ hearts relative to WT counterparts (Figure 4B and Supplemental Table 3). Interestingly, many of these differentially expressed proteins at both ages were mitochondrial proteins, with functional roles in oxidative phosphorylation (Figure 4, A and C). Clustering analysis, using the STRING database of known and predicted protein interaction networks, was performed to investigate if the differentially expressed proteins clustered around molecular and functional networks. Interestingly, *ECSIT*^+/+^ heart samples demonstrated increased expression of 26 and decreased expression of 34 mitochondrial proteins at 3 months of age. While there was a lack of functional clustering of proteins of increased expression (beyond their mitochondrial localization), the profile of proteins of reduced expression was intensely clustered around complex I of the mitochondrial electron transport chain (Figure 4D). At 10 months of age, the number of mitochondrial proteins of increased expression in *ECSIT*^+/+^ heart increased to 99, including cytochrome C oxidase (Cox) subunits of complex IV and subunits of the ATP synthase. The number of mitochondrial proteins of decreased abundance at 10 months in *ECSIT*^+/+^ heart was 40, and these again strongly clustered around complex I (Figure 4D). The population of decreased proteins at 10 months also clustered around a network of contractile proteins, such as actin, actin-binding proteins (e.g., tropomyosins), and myosin proteins. The findings from the proteomic analysis of heart tissue were confirmed by Western blotting of cardiac tissue and validating that levels of complex I subunits such as NADH dehydrogenase [ubiquinone] iron-sulfur protein 3 (NDUFS3) were greatly decreased, while Cox IV and Cox V proteins were slightly increased in *ECSIT*^+/+^ heart (Figure 4E). Other mitochondrial proteins, such as the outer membrane protein Tom20, were present in comparable amounts in WT and *ECSIT*^+/+^ heart. Given that *ECSIT*^+/+^ heart displayed a signature proteomic profile of greatly reduced levels of many complex I subunits, we next compared the activities of complex I in 7-month-old WT and *ECSIT*^+/+^ heart using Blue Native polyacrylamide gel electrophoresis (BN-PAGE) in conjunction with in-gel activity assay and a specific complex I substrate. The findings clearly demonstrate that heart tissue from 7-month-old *ECSIT*^+/+^ mice had greatly impaired activity of the individual complex I as well as marked reductions in activity of complex I as part of higher order supercomplexes (Figure 4F). Interestingly such impaired complex I activity was also observed in younger 10-week-old *ECSIT*^+/+^ mice (Figure 4G), suggesting that complex I deficiency may precede the development of cardiac hypertrophy. In contrast, equivalent analysis demonstrates the same level of activity of complex IV as an individual complex and as part of a supercomplex with complex III in WT and *ECSIT*^+/+^ heart, suggesting that reduced levels of ECSIT specifically impair the function of complex I (Figure 4, F and G). These data provide valuable insight into the molecular basis underlying the cardiac hypertrophy observed in *ECSIT*^+/+^ mice. In young mice at 3 months old, the cohort of proteins of decreased expression were mostly subunits of complex I, with the primary defect in *ECSIT*^+/+^ heart in early life being reduced complex I activity. This will lead to a compromise of mitochondrial and ATP production that will likely lead to adaptive and compensatory response, such as hypertrophy. The defect in complex I continued to be observed at 10 months of age, but the population of proteins of decreased expression expanded to include contractile proteins that play fundamental roles in cardiac muscle contraction, and such effects on contractile proteins, in conjunction with increased fibrosis, is likely a prelude to cardiac failure and premature death.

### Heart from human ECSIT–knockin mice has fragmented mitochondria characterized by greater fission and reduced fusion.

**Figure 5:**
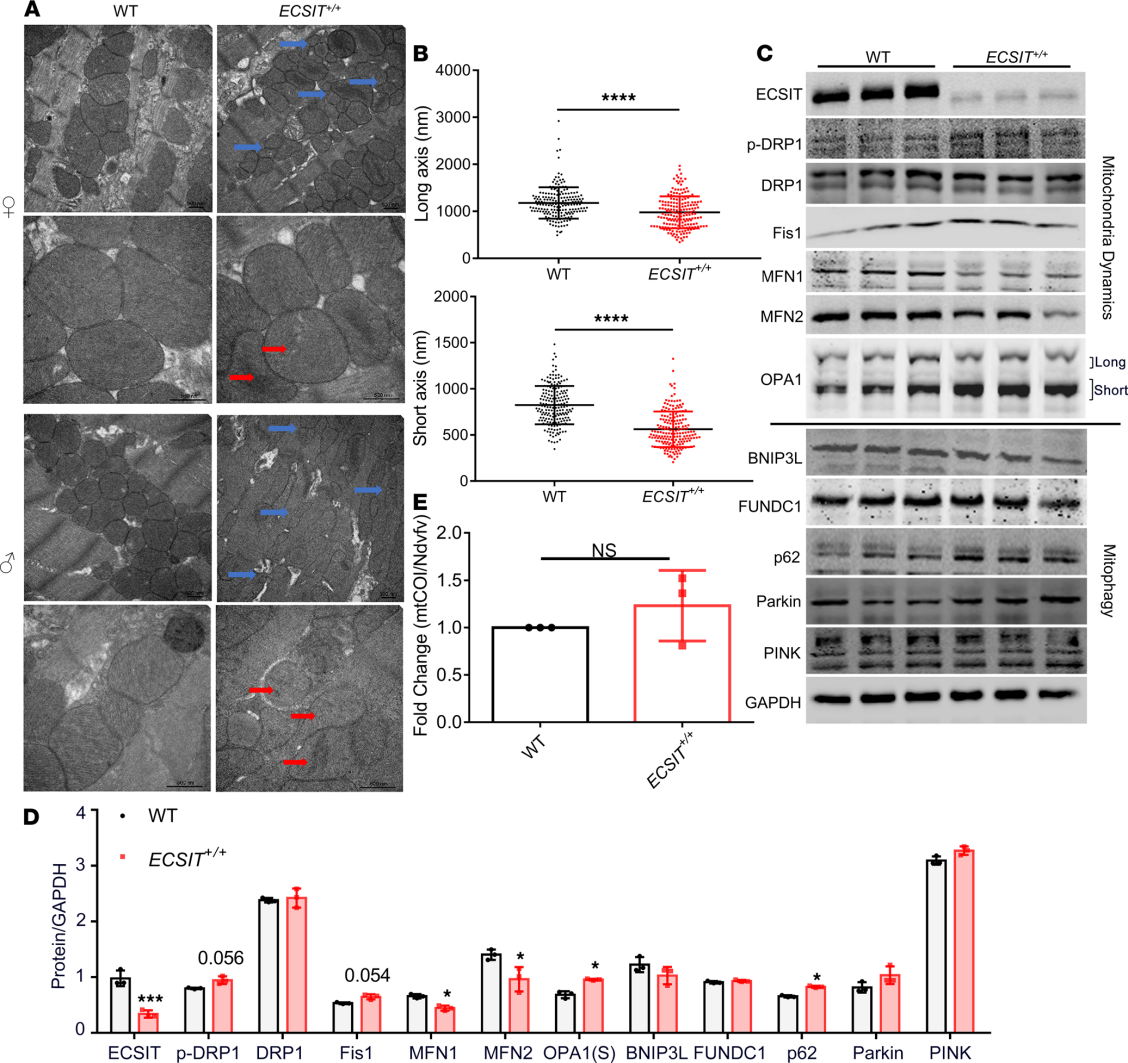
Hearts from human ECSIT–knockin mice show increased fission and fragmented mitochondria. (**A**) Electron microscopy of the left ventricular tissue from 6-month-old female and 7-month-old male WT and *ECSIT^+/+^* mice (scale bars: 500 nm). Red arrows indicate mitochondria with distorted or absent cristae. Blue arrows indicate small and fragmented mitochondria. (**B**) Mean long and short axes’ lengths (nm) of mitochondria (*n* = 190) in WT and *ECSIT^+/+^* mice. (**C**) Immunoblot analysis of left ventricular cardiac tissue for levels of ECSIT, phosphorylated DRP1 (p-DRP1), DRP1, Fis1, mitofusin 1 (MFN1), mitofusin 2 (MFN2), OPA-1, p62, Parkin, PTEN-induced kinase 1 (PINK-1), and GAPDH in WT and *ECSIT^+/+^* mice of 8–10 weeks of age (*n* = 3). (**D**) Quantitative analysis of mitochondria dynamics and mitophagy proteins (*n* = 3, individual mouse hearts). (**E**) Ratio of mitochondrial DNA encoding COI/nuclear DNA encoding ndufv1 as determined by quantitative PCR of cardiac tissue from 7-month-old WT and *ECSIT^+/+^* mice (*n* = 3, individual mouse hearts). (**B**, **D**, and **E**) Data are expressed as the mean ± SD from 3 independent experiments; unpaired 2-tailed Student’s *t* test. *P < 0.05, ***P < 0.001, ****P < 0.0001.

Electron microscopy was next performed on tissue from the left ventricles of WT and *ECSIT*^+/+^ mice in order to understand the consequences of impaired mitochondrial complex I at the ultrastructural level. Tissue from WT mice showed mitochondria in the characteristic highly ordered state along the sarcomeres whereas the arrangement of mitochondria in tissue from *ECSIT*^+/+^ mice was less ordered and more irregular (Figure 5A). Such disorder was apparent in heart tissue from male and female mice. Mitochondria from *ECSIT*^+/+^ tissue also showed distorted cristae, with some mitochondria exhibiting total or partial absence of cristae (highlighted by red arrow in Figure 5A). We also noted that the size of mitochondria from *ECSIT*^+/+^ tissue was more heterogeneous than in WT mice, with some of the mitochondria appearing much smaller and rounded than the rest of the population (highlighted by blue arrows in Figure 5A). Quantitative analysis of multiple images confirmed that the mean lengths of both the long and short axes of mitochondria were shorter in *ECSIT*^+/+^ tissue (Figure 5B). The presence of smaller mitochondria in *ECSIT*^+/+^ heart suggested potential differences in mitochondrial dynamics between WT and *ECSIT*^+/+^ mice. The quality of mitochondria is generally controlled by a dynamic process in which asymmetric fission, mediated by Dynamin related protein 1 (Drp-1) and mitochondrial fission 1 protein (Fis1), separates a damaged mitochondrion into a depolarized daughter organelle that can be targeted for removal by mitophagy (25), with the other daughter component fusing to the functional mitochondrial pool by the action of the fusion proteins mitofusin 1 and mitofusin 2 in the outer mitochondrial membrane and the fusion protein optic atrophy 1 (Opa-1) in the inner membrane (26). Western blotting of cardiac tissue from WT and *ECSIT*^+/+^ mice showed that levels of Fis1 and phosphorylated Drp-1 were increased in *ECSIT*^+/+^ tissue whereas levels of the fusion proteins mitofusin 1 and 2 were reduced with concomitant processing of Opa-1 (Figure 5, C and D), all consistent with *ECSIT*^+/+^ tissue showing greater fission and impaired fusion. Since fission generates fragmented mitochondria that can be removed by mitophagy, we were keen to compare levels of mitophagy markers in WT and *ECSIT*^+/+^ tissue. *ECSIT*^+/+^ tissue showed slightly augmented levels of p62 and Parkin but no PINK accumulation (Figure 5, C and D), suggestive of some interference or saturation of the mitophagy process that would normally promote their lysosomal-mediated degradation. Parkin-independent mitophagy mediated by BCL2-interacting protein 3 like (BNIP3L) and FUN14 domain–containing 1 (FUNDC1) receptors was comparable in WT and *ECSIT*^+/+^ heart tissue (Figure 5, C and D). The enhanced mitochondrial fission without concomitant increase in mitophagy observed in *ECSIT*^+/+^ heart tissue is fully consistent with our ultrastructural electron microscopy analysis showing the accumulation of smaller fragmented mitochondria in these hearts. While *ECSIT*^+/+^ heart tissue was characterized by the presence of some fragmented mitochondria, the overall mitochondrial content, at least as measured by mitochondrial genome markers, was comparable to WT heart tissue (Figure 5E). However, overall mitochondrial function was likely compromised given that fragmented mitochondria are frequently damaged and impairing function may also contribute to disease by production of ROS.

### Suppressed expression of hECSIT in cardiomyocytes impairs complex I activity and leads to cellular hypertrophy.

**Figure 6:**
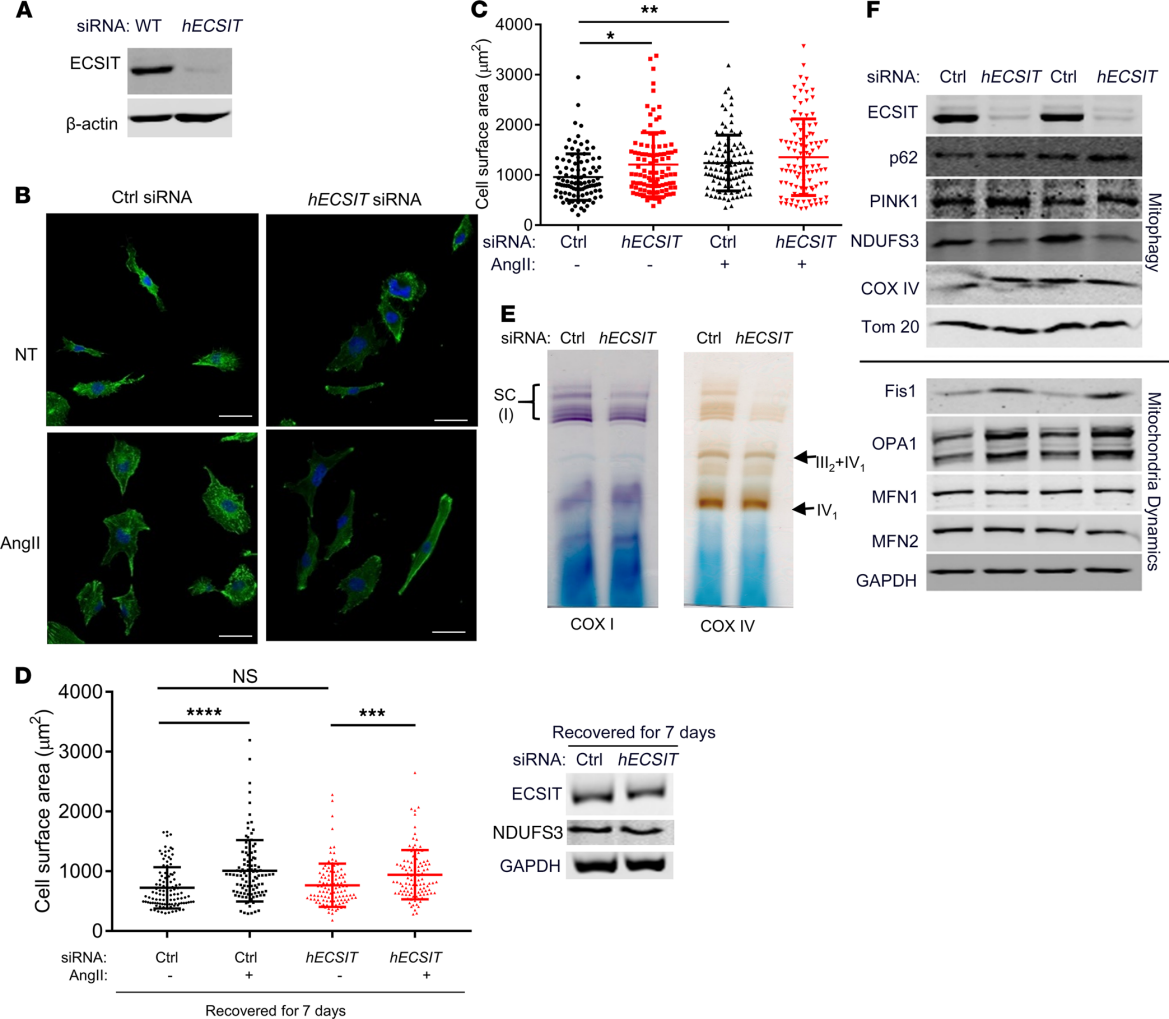
Knockdown of hECSIT expression in cardiomyocytes impairs mitochondrial complex I activity and promotes cellular hypertrophy. (**A**) Immunoblot analysis, using anti-ECSIT and anti–β-actin antibodies, of cell lysates from AC16 cardiomyocyte cells transfected with control (Ctrl) or *hECSIT*-specific siRNA (40 nM). (**B** and **C**) AC16 cardiomyocytes transfected with control or *hECSIT*-specific siRNA and then treated with PBS or Angiotensin II (Ang II; 2 μM) for 24 hours. (**B**) Cells were stained for α-Actinin staining by immunofluorescence microscopy with nuclei being visualized by DAPI staining. Scale bars: 50 μm. (**C**) Quantification of cell surface area of AC16 cells (100 cells were measured for each treatment from 3 independent experiments). (**D**) Quantification of cell surface area of AC16 cells (100 cells were measured for each treatment from 2 independent experiments) transfected with control or *hECSIT*-specific siRNA, allowed to recover and grown for 7 days, and then treated with PBS or Angiotensin II (Ang II; 2 μM) for 24 hours. Immunoblot analysis, using anti-ECSIT, anti-NDUFS3, and anti-GAPDH antibodies, of cell lysates. (**E**) Isolated heart mitochondria from AC16 cells, previously transfected with control or *hECSIT*-specific siRNA, were subjected to BN-PAGE followed by in-gel activity assays using complex I– and complex IV–specific substrates. Complex I activity, as part of supercomplexes (SC), is shown in violet. Complex IV activity, as an individual complex or as part of supercomplex with complex III, is shown in brown. (**F**) Immunoblot analysis of lysates from AC16 cells, previously transfected with control or *hECSIT* specific siRNA, for levels of ECSIT, p62, PINK1, NDUFS3, Cox IV, Tom20, Fis1, mitofusin 1 (MFN1), mitofusin 2 (MFN2), OPA-1, and GAPDH. Data represent at least 2 biological replicates. (**C** and **D**) Data are expressed as the mean ± SD; 1-way ANOVA with Dunnett’s multiple comparisons test; *P < 0.05, **P < 0.01, ***P < 0.001, ****P < 0.0001.

Given that hECSIT expression is suppressed in a number of tissues from *ECSIT*^+/+^ mice, its direct role in cardiomyocytes was next examined in order to assess if its cell-intrinsic function in cardiac cells may underlie the mitochondrial dysfunction and cardiac hypertrophy observed in *ECSIT*^+/+^ mice. We were also keen to show that the latter effects in *ECSIT*^+/+^ mice were due to reduced levels of hECSIT and not due to differences in function for hECSIT and mEcsit. To this end, we used hECSIT-specific siRNA to suppress endogenous expression of hECSIT in human AC16 cardiomyocytes (Figure 6A). Immunofluorescence staining of hECSIT-knockdown cells showed an apparent increase in the overall cell size (Figure 6B), and this was confirmed by quantitative analysis of cell surface area (Figure 6C). Such hypertrophy was reversible since it was not apparent when hECSIT-specific siRNA–knockdown cells were grown for an additional 7 days to recover from transient siRNA-mediated downregulation of hECSIT (Figure 6D). The magnitude of cell hypertrophy induced by knockdown of hECSIT was comparable to that observed when cells were treated with Angiotensin II, a well-known driver of cardiac hypertrophy (Figure 6C). Thus reduced expression of hECSIT in human cardiomyocytes that leads to cellular hypertrophy mirrors at a cellular level the cardiac hypertrophy observed in the *ECSIT*^+/+^ mice that express low levels of hECSIT. Furthermore, suppression of hECSIT in cardiomyocytes also shows the same effects on mitochondrial function as described in cardiac tissue from *ECSIT*^+/+^ mice in that BN-PAGE analysis revealed that knockdown of hECSIT in AC16 cells impaired the activity of complex I as part of mitochondrial supercomplexes whereas other complexes, such as complex IV, were unaffected (Figure 6E). Such impaired complex I activity in hECSIT-knockdown AC16 cells is also consistent with reduced levels of complex I subunits, as typified by NDUFS3, whereas complex IV protein and other mitochondrial proteins like Tom20 were not affected by suppression of hECSIT (Figure 6F). Knockdown of hECSIT also resulted in modest increases in levels of p62 and PINK-1. Similar to *ECSIT*^+/+^ cardiac tissue, hECSIT-knockdown AC16 cells showed signs of increased mitochondrial fission and reduced fusion as indicated by enhanced levels of the fission protein Fis1 and increased processing of the fusion protein (Opa-1; Figure 6F). Notably the levels of mitofusin 1 and 2 were not affected by knockdown of hECSIT, suggesting that additional factors may be required to manifest their reduced expression in *ECSIT*^+/+^ cardiac tissue. These data show that threshold levels of hECSIT are required for normal mitochondrial function and dynamics in cardiomyocytes, with reduction below these levels resulting in compensatory changes, such as cellular hypertrophy. This closely mirrors the cardiac pathophysiology observed in *ECSIT*^+/+^ mice that express low amounts of hECSIT.

### Reduced levels of hECSIT promote a metabolic shift from oxidative phosphorylation to glycolysis.

**Figure 7:**
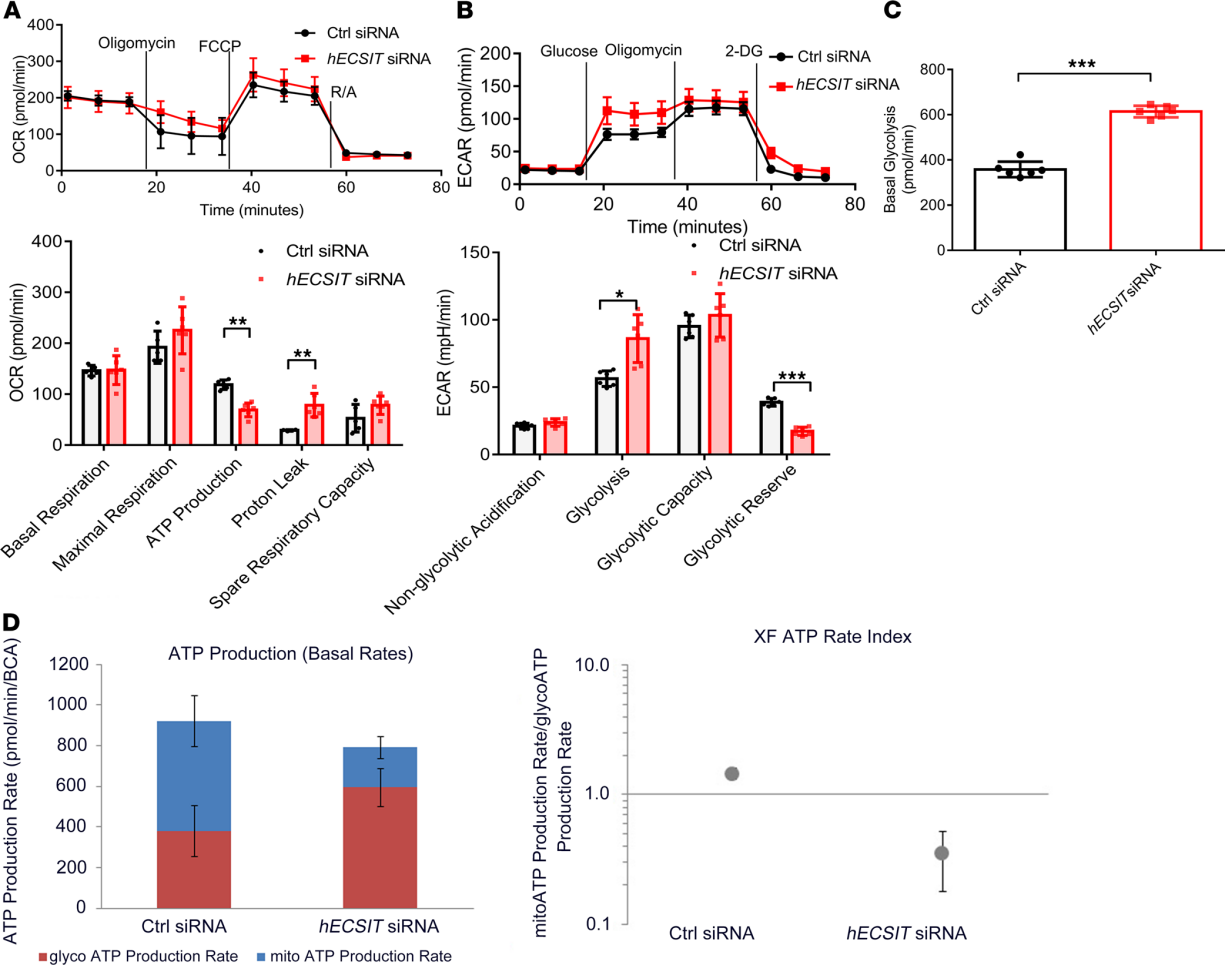
Reduced levels of hECSIT cause a metabolic shift from oxidative phosphorylation to glycolysis. (**A**) Analysis of OCR in AC16 cells transfected with control and *hECSIT* siRNA to assess rates of basal respiration, maximal respiration, respiration linked to ATP production, proton leak, and spare respiratory capacity. (**B**) Analysis of extracellular acidification rate (ECAR) in AC16 cells transfected with control and *hECSIT* siRNA to assess nonglycolytic acidification, glycolysis, glycolytic capacity, and glycolytic reserve. (**C**) Glycolysis-driven proton efflux rate in AC16 cells transfected with control and *hECSIT* siRNA. (**D**) Rates of ATP production as mediated by glycolysis or mitochondrial metabolism in AC16 cells transfected with control and *hECSIT* siRNA (*left panel*). Ratio of ATP produced by oxidative phosphorylation/ATP produced by glycolysis ATP in AC16 cells transfected with control and *hECSIT* siRNA (*right panel*). Data are expressed as the mean ± SD from 4 to 6 technical replicates, and each experiment has at least 2 biological replicates; unpaired 2-tailed Student’s *t* test applied. *P < 0.05, **P < 0.01, ***P < 0.001.

**Figure 8:**
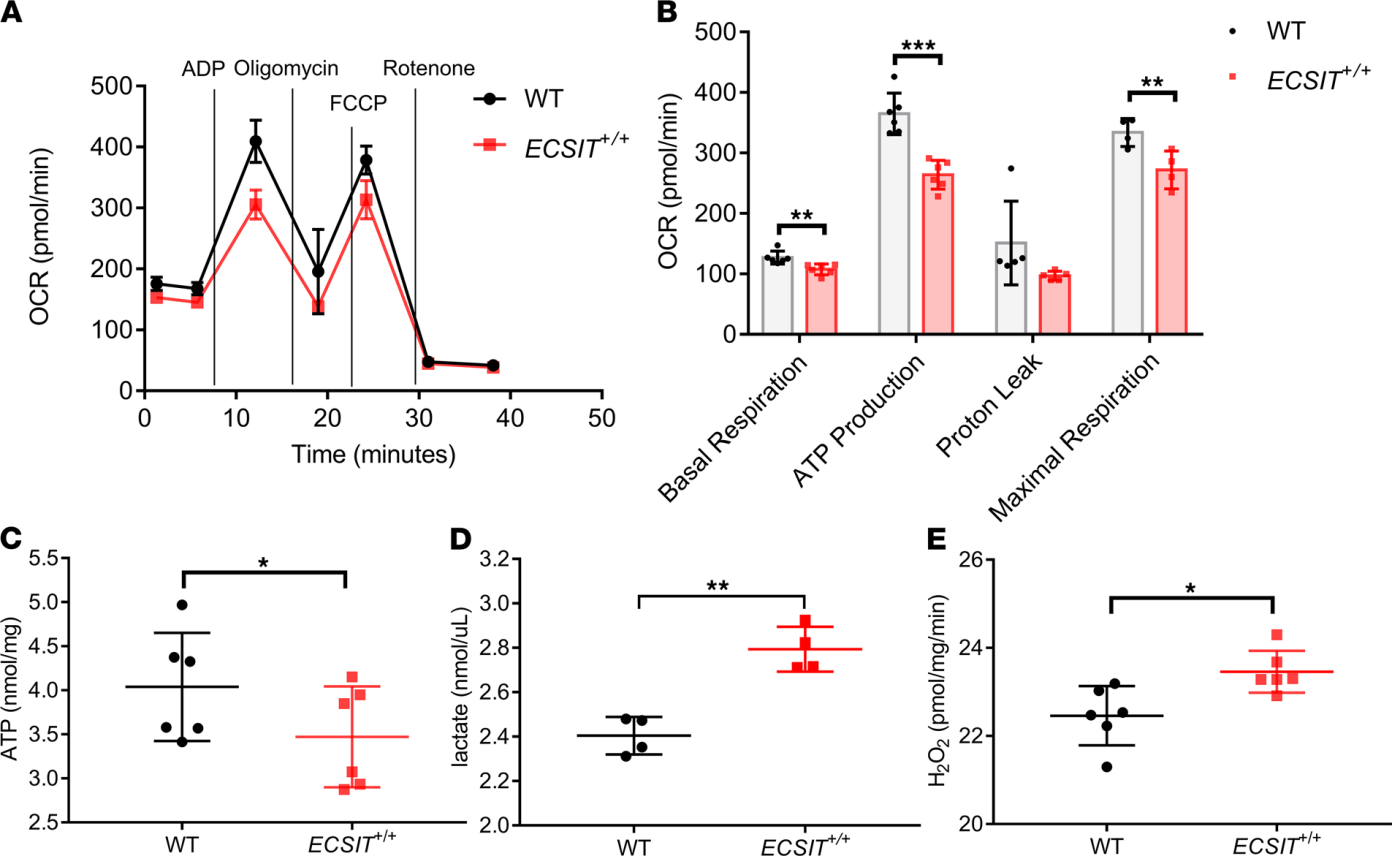
Reprogramming of primary metabolism to glycolysis in humanized hECSIT heart. (**A**) Analysis of OCR in mitochondria isolated from WT and *ECSIT^+/+^*. (**B**) Quantitative data of basal respiration, maximal respiration, ATP production, and proton leak in mitochondria isolated from WT and *ECSIT^+/+^*. (**C**) Levels of ATP in left ventricular tissue from hearts of WT and *ECSIT^+/+^* mice 6–7 months old. Data are expressed as the mean ± SD (*n* = 6, 2 pair mice, 3 technical repeats for each pair, sex mixed); *P < 0.05, paired 2-tailed Student’s *t* test. (**D**) Levels of lactate in left ventricular tissue from hearts of WT and *ECSIT^+/+^* mice (*n* = 4) 7 months old. Data are expressed as the mean ± SD. (**E**) Levels of H_2_O_2_ in left ventricular tissue from hearts of WT and *ECSIT^+/+^* mice 6 to 7 months old. Data are expressed as the mean ± SD (*n* = 6, 2 pair mice, 3 technical repeats for each pair, sex mixed); *P < 0.05, unpaired 2-tailed Student’s *t* test. (**A** and **B**) Data are expressed as the mean ± SD from 4–6 technical replicates, and each experiment has 2 biological replicates; unpaired 2-tailed Student’s *t* test applied. *P < 0.05, **P < 0.01, ***P < 0.001.

Given that the knockdown of hECSIT in AC16 cardiomyocytes models many of the pathophysiological features in hearts from *ECSIT*^+/+^ mice, this model was used to better understand how the role of hECSIT in mitochondrial function contributes to cardiac physiology. Since cardiac hypertrophy is frequently a response to a deficit in serving the enormous bioenergetics requirements of the heart and mitochondria are the major producers of ATP in cardiomyocytes to satisfy this energy demand, we next assessed the role of hECSIT in primary metabolism and respiration in cardiomyocytes. To this end, the effect of hECSIT knockdown in AC16 cardiomyocytes on metabolic flux analysis was investigated. Oxygen consumption rate (OCR) was measured to characterize the effect of hECSIT knockdown on the various contributory pathways to respiration. While basal respiration, maximal respiration, and spare respiratory capacity were the same in AC16 cells transfected with control or hECSIT-specific siRNA, the OCR that was associated with ATP production was significantly diminished in hECSIT-knockdown cells (Figure 7A). Maximal respiration was not affected since the shortfall in respiration associated with ATP production was compensated by increased respiration due to proton leak. The decreased respiration associated with ATP production translates to reduced levels of oxidative phosphorylation, and metabolic flux analysis was next applied to assess whether hECSIT-knockdown cells demonstrated a reprogramming to the glycolytic pathway, which was characterized by measuring the extracellular acidification rates (ECARs) in control and hECSIT-knockdown cells. AC16 cells, transfected with hECSIT-specific siRNA, demonstrated a tendency to an increased ECAR in glycolysis (Figure 7B), with a more precise measurement of glycolytic rate confirming that hECSIT knockdown resulted in increased rates of basal glycolysis (Figure 7C). The overall glycolytic capacity of the cells was not affected, and as expected the increased rate of glycolysis was associated with a decrease in the glycolytic reserve (Figure 7B). These data clearly indicate that reduced expression of hECSIT in cardiomyocytes results in reduced levels of oxidative phosphorylation with a metabolic reprogramming to increased rates of glycolysis. Metabolic flux also allowed for calculation of the amounts of ATP generated by these pathways and confirmed that hECSIT-knockdown cells manifested higher rates of ATP production by glycolysis and less ATP by oxidative phosphorylation than control cells (Figure 7D, *left panel*). Indeed the magnitude of this shift is highlighted by the ratio of ATP produced by oxidative phosphorylation/ATP produced by glycolysis decreasing from 1.45 in control cells to 0.35 in hECSIT-knockdown cells (Figure 7D, *right panel*). These findings are consistent with a model in which reduced levels of hECSIT in knockdown cells leads to impairment of complex I activity and reduced oxidative phosphorylation and ATP production, resulting in a compensatory shift to ATP production by increased rates of glycolysis. The pathophysiological reliance of this metabolic programming in response to reduced levels of ECSIT was supported by similar metabolic patterns in isolated mitochondria from *ECSIT*^+/+^ hearts. Thus *ECSIT*^+/+^ mitochondria showed reduced oxygen consumption (Figure 8A), impairment of basal and maximal respiration (Figure 8B), and reduced rates of ATP production (Figure 8B) relative to isolated mitochondria from WT mice. In addition cardiac tissue from *ECSIT*^+/+^ mice showed reduced levels of ATP (Figure 8C). This reflects a reprogramming of primary metabolism to increased glycolysis that was confirmed by increased levels of the glycolytic end product lactic acid in cardiac tissue from *ECSIT*^+/+^ mice (Figure 8D). The reduction in oxidative phosphorylation was also associated with incomplete reduction of oxygen to form ROS products as measured by increased levels of H_2_O_2_ (Figure 8E). The compensatory shift to glycolysis clearly did not fully restore the ATP-generating capacity of cardiomyocytes, and in the in vivo setting this likely led to an adaptive cardiac hypertrophy response that we observed in *ECSIT*^+/+^ mice.

All of these data show that the levels of hECSIT are a critical determinant of mitochondrial function, and we now provide the first insight to our knowledge into the key regulatory processes that dictate the stability of this protein.

## Discussion

Prior to this study, a number of functions had already been ascribed to Ecsit proteins, including an indispensable role of mEcsit in BMP signaling in early development, as a positive regulator of the NF-κB pathway and inflammatory gene expression, and as an important factor in promoting assembly of complex I in the electron transport chain of the mitochondria (5, 6, 11, 15). Furthermore, mEcsit was also reported to facilitate the juxtaposition of phagosomes and mitochondria to enhance mROS production and bactericidal activity in response to *Salmonella* infection (12). Exploiting the labile nature of hECSIT, we now show that whereas early developmental pathways, activation of NF-κB, and induction of proinflammatory cytokines are all functionally intact with even low levels of hECSIT, the mitochondrial functions of the latter are critically dependent on its expression levels. In the process we also develop a mouse model that reveals what we believe is the first physiological role for ECSIT in an in vivo setting. We describe the critical contribution of hECSIT to cardiac physiology with threshold levels of its expression being required to ensure normal cardiac function. We show that subthreshold levels of hECSIT contribute to cardiac disease as revealed by the manifestation of cardiac hypertrophy in *ECSIT*^+/+^ mice and the association of low levels of hECSIT with myocardial hypertrophy and cardiac fibrosis in human cohorts. It should be noted that due to lack of availability of human tissue from the left ventricle, fibrosis measurements were performed on atrial appendages that were available from clinically annotated cohorts. While hECSIT plays a fundamental role in mitochondrial function, it is interesting to note that low levels of hECSIT primarily manifest in cardiac dysfunction, leading to cardiac hypertrophy without showing any other obvious effects on the other main organs. This may be due to the extremely high demands of heart requiring highly efficient electron transport and oxidative phosphorylation for ATP production with any deficiencies in these processes, such as impaired complex I under conditions of low hECSIT expression, resulting in essential compensatory processes and adaptation as occurs in cardiac hypertrophy. The lower energy demands of other tissues may be more tolerant of loss of complex I and mitochondrial function. Given that expression levels of hECSIT appear to be critical for cardiac physiology, it is interesting to speculate on why evolutionary pressure would not select against hECSIT losing its protein stability whereas in other mammals such as mice, mECSIT is significantly more stable. There are significant differences in cardiac physiology in mice and humans. The high workload of the heart places major metabolic and energy demands on this organ. This is especially relevant to mice that have average heartbeats of 600/min whereas the average heartbeat in humans is less by an order of magnitude. Thus, there is strong pressure in mice to maximize mitochondrial output whereas this need also applies in human hearts but may not be subject to the extreme demands of mouse hearts. Indeed, while the stability of hECSIT is adequate for human demands, the phenotype of cardiac hypertrophy in humanized hECSIT-knockin mice demonstrates that its stability is not sufficient to cater to the energy demands in mouse, thus promoting compensatory and adaptive processes such as hypertrophy. A recent report has also highlighted a potential evolutionary pressure that may favorably select the labile nature of hECSIT. Thus a recurrent and hyperactive ECSIT mutation, which results in greatly augmented downstream activation of NADPH and NF-κB and induction of proinflammatory cytokines, can trigger the fatal hyperinflammatory disease hemophagocytic syndrome (7). The labile nature of hECSIT is likely to exercise tighter control over its downstream inflammatory effects and so minimize the risk of hyperinflammation. Notably, embryonic lethality, caused by *mEcsit* deficiency, is rescued by knockin of *hECSIT*, indicating that the human ortholog can serve the same developmental role in the BMP pathway. Interestingly, no hECSIT mutations have been reported to date, and this may reflect that total loss of function is incompatible with life. However, mutations in another factor, ACAD9, which also associates with NDUFAF1 and ECSIT, appear to be a relatively common cause of complex I deficiency presenting as cardiomyopathy and/or exercise intolerance (10).

We also map out the cellular and molecular basis to the critical role of hECSIT in normal cardiac function. Thus, hECSIT is essential for assembly and maintenance of mitochondrial complex I and driving efficient oxidative phosphorylation and ATP production in cardiomyocytes. Low-level expression of hECSIT caused reduction in oxidative phosphorylation, and this likely led to dysfunctional and depolarized mitochondria. Indeed the heart tissue from *ECSIT*^+/+^ mice showed some fragmented mitochondria associated with increased fission and reduced fusion. Normally fission is a prologue to removal of damaged mitochondria by mitophagy. Instead mitophagy was not effectively or sufficiently activated in *ECSIT*^+/+^ mice, and this likely led to the accumulation of damaged fragmented mitochondria that was observed by electron microscopy. Limited mitophagy may be due to the reduced levels of mitofusin 2, a receptor for Parkin, or may be a more direct consequence of reduced levels of hECSIT in light of a recent report describing a role for mEcsit in mitophagy by acting as a substrate for Parkin (14). In response to reduced oxidative phosphorylation under conditions of low hECSIT, there is a metabolic shift to glycolysis to cater for reduced production of ATP. However, this switch does not fully compensate for the reduced ATP production, and this likely underlies the ensuing hypertrophy that is likely to be adaptive at the early stage but over time progresses to a pathological situation and heart failure.

There is a close association of mitochondrial dysfunction with cardiac disease, but the molecular basis to this relationship is not fully delineated. Given that deficiencies in complex I are a very common defect in mitochondrial diseases, including heart failure and cardiomyopathy, there is much interest in gaining a better understanding of disease progression and new treatments. The lack of good models has contributed to the slow progress in this regard. We have now developed a model that may be very valuable in dissecting the pathophysiology of cardiac disease progression that is associated with complex I and mitochondrial dysfunction and in evaluating the preclinical therapeutic potential of new lead candidates for disease treatment. The model offers a number of attractive features. First, the cardiac hypertrophy associated with the model develops gradually over time, and this may have application in modeling the slow progression of human cardiac dysfunction with age. This may complement and indeed surpass many of the widely used mouse models that tend to use relatively acute pressure overload to induce cardiac pathology but may not capture the mechanisms underlying the more gradual pathogenesis in humans. Interestingly, a recent study has reported the expression of hECSIT to decrease with age (27) and in the context of our present findings, this may have important consequences for cardiac disease as part of the aging process. Other genetic models have also been generated to study cardiac hypertrophy and disease. These include a transgenic mouse overexpressing myotropin, which stimulates myocyte growth (28), and a knockout mouse lacking cardiac myosin binding protein-C (cMyBP-C; ref. 29). While these models have been valuable in terms of studying cardiac disease, the interpretation of data from such mice is complicated by cardiac disease developing very early, with the cMyBP-C knockout showing cardiac hypertrophy and remodeling during perinatal development. The slow and gradual development of cardiac hypertrophy in the *ECSIT*^+/+^ mice offers much promise to address this current limitation in animal models to study cardiac disease progression. Furthermore, the reduced levels of most of the protein subunits in mitochondrial complex I in *ECSIT*^+/+^ mice offers opportunity to better understand the role of complex I in a number of diseases. Deficiencies in mitochondrial complex I in humans manifest as diseases ranging from hypertrophic cardiomyopathy to liver disease and neuropathological conditions, including Parkinson’s disease. There is a paucity of genetic models of complex I deficiency that allow for study of slow disease progression since deficiency in complex I subunits tends to lead to developmental lethality or death at very young ages. Here, we generate humanized hECSIT mice with complex I deficiency but with a life span of approximately 1 year. These mice may serve as a valuable resource in future study of complex I–related diseases.

In summary these studies describe the physiological role for hECSIT in cardioprotection and in the process develop a model to study disease progression in mitochondria dysfunction and heart disease.

## Methods

Further information can be found in Supplemental Methods.

### Mice.

Humanized mice were generated by Taconic Artemis using proprietary technology. To generate human *ECSIT*–knockin mice, heterozygotes (*ECSIT^+/–^*) for the targeted allele were bred with mice containing a *Flpe* transgene, C57BL/6N-Tg(CAG-Flpe)2Arte. This resulted in the replacement of exons 3 to 9 of murine *ECSIT* with exons 2 to 8 of human *ECSIT*. The *Flpe* transgene was removed by breeding the resulting mice with C57BL/6N mice during colony expansion. Mice were genotyped by PCR analysis of DNA isolated from ear punches using primers “a” and “b.” All animals were used at age 8 to 12 weeks unless indicated otherwise. Animals were allocated to experimental groups based on genotype, age, and sex. No randomization methods were used.

### Mitochondria isolation.

Mouse hearts (2 pairs 7 months old, sex mixed) or cells (AC16) were washed with ice-cold washing buffer (10 mM HEPES pH 7.2 containing 0.3 M sucrose,0.2 mM EDTA), minced, and homogenized in isolation buffer (10 mM HEPES pH 7.4 containing 0.3 M sucrose, 0.2 mM EDTA, 1 mg/mL BSA, 1× trypsin-EDTA solution). After the homogenate was centrifuged at 4°C for 15 minutes at 600*g*, the supernatant was collected and further centrifuged at 4°C for 15 minutes at 8500*g*. The pellet was resuspended for functional assessment. The protein concentration of the mitochondrial preparation was determined by BCA assay (Thermo Fisher Scientific).

### Blue native polyacrylamide gel electrophoresis (BN-PAGE).

Blue NativePAGE was conducted using the NativePAGE system (Invitrogen). Briefly, isolated cardiac mitochondria (50 μg) were solubilized in 20 μL of NativePAGE sample buffer supplemented with 2% (*w/v*) digitonin for 20 minutes on ice, then centrifuged at 20,000*g* at 4°C for 10 minutes. After centrifugation, the supernatants were collected. Coomassie blue G-250 (Invitrogen) was added to the supernatant to obtain a dye/detergent mass ratio of 4:1, and then the protein was loaded into a NativePAGE 3% to 12% gradient gel (Invitrogen). The gel was run at 150 V for 30 minutes, and then the light blue buffer in the inner chamber was refreshed with 200 mL of cathode buffer. The gel was run at 250 V for 150 minutes.

For in-gel assay of complex I/IV activities, gels were incubated in 10 mL of complex I substrate solution (2 mM Tris-HCl pH 7,4 containing 0.1 mg/mL NADH, 2.5 mg/mL Nitrotetrazolium Blue chloride) or complex IV substrate solution (0.5 mg/mL diaminobenzidine and 1 mg/mL cytochrome c in 45 mM phosphate buffer pH 7.4). Complex I and complex IV activities manifested as violet and brown bands, respectively. Reactions were terminated with 10% (v/v) acetic acid followed by washing with water and images captured by scanning.

### Lactate assay.

Left ventricular tissue from 6 to 7 months mice (*n* = 4, 2 pairs male and 2 pairs female) was homogenized in RIPA buffer: 50 mM HEPES pH 7.5, 10% (v/v) glycerol, 0.5% (*w/v*) CHAPS, 0.5% (v/v) Triton X-100, 150 mM NaCl, 1 mM Na_3_VO_4_, 1 mM EDTA, 1 mM PMSF, and complete protease inhibitor mixture. Protein concentration was determined by BSA assay, and perchloric acid was added to a final concentration of 1 M to precipitate protein. Supernatants were neutralized by adding ice-cold 2 M KOH to reach pH 6.5–8.0. To measure l-lactate, we used l-lactate assay kit (MAK064-1KT, MilliporeSigma).

### Mitochondrial mass.

For mitochondrial mass determination, genomic DNA was extracted from tissues (*n* = 3, around 7 months old, male) using the DirectPCR Lysis reagent (102-T, Viagen Biotech), following the manufacturer’s instructions. DNA concentrations were determined by NanoDrop (Thermo Fisher Scientific). Ten nanograms DNA was used to perform quantitative PCR for mitochondrial gene mtCOI and nuclear gene ndufv1. Ratios of 2^–ΔCT^ for mtCOI over ndufv1 were averaged and fold of WT is shown. The primers used are in Supplemental Table 4.

### RNA interference.

Human-specific *ECSIT* (4390825, Assay ID s224197) and control siRNAs (4390844) were purchased from Thermo Fisher Scientific. siRNA (40 nM) was delivered to AC16 via transfection with Lipofectamine RNAiMAX Transfection Reagent (13778030) according to the manufacturer’s instructions and allowed to recover for 48 hours prior to experiments.

### Metabolic flux analysis of cells.

Cell metabolism was measured using a Seahorse XF96 Extracellular Flux Analyzer according to the manufacturer’s instructions. Briefly, AC16 cells (pretreated with control or *hECSIT* siRNA) were seeded onto an XF96 microplate at 50,000 cells/well. The cellular OCR used in the mitochondria stress test was monitored in seahorse assay medium (catalog 103575-100) supplemented with 2 mM glutamine, 1 mM sodium pyruvate, and 25 mM glucose (pH 7.4 at 37°C), following the sequential addition of oligomycin (2 μM), FCCP (2 μM), and rotenone (1 μM) and antimycin A (AA) (0.5 μM). ECAR used in the glycolysis stress test was monitored in seahorse assay medium (catalog 103575-100) supplemented with 2 mM glutamine, (pH 7.4 at 37°C), following the sequential addition of glucose (10 mM), oligomycin (2 μM), 2-DG (50 mM). Glycolysis proton efflux rates (glycoPER) used in the glycolytic rate was monitored in seahorse assay medium supplemented with 2 mM glutamine, 10 mM glucose, 1mM pyruvate, (pH 7.4 at 37°C), following the sequential addition of rotenone (1μM) and AA (0.5 μM), and 2-DG (50 mM). Real-time ATP rate was monitored in seahorse assay medium supplemented with 2 mM glutamine, 10 mM glucose, and 1 mM pyruvate, (pH 7.4 at 37°C), following the sequential addition of oligomycin (2 μM), rotenone (1 μM), and AA (0.5 μM).

### Metabolic flux analysis of isolated mitochondria.

Mitochondrial bioenergetics in isolated mitochondria from hearts was determined as described previously (30). Hearts from 10-week-old mice were rinsed and isolated in MIB1 buffer (210 mM d-Mannitol, 70 mM sucrose, 5 mM HEPES, 1 mM EGTA, and 0.5% fatty acid–free BSA, pH 7.2). Isolated mitochondria were seeded onto an XF96 microplate at 6 μg/well. Mitochondrial assay was performed in MAS1 buffer (220 mM d-Mannitol, 70 mM sucrose, 10 mM KH_2_PO_4_, 5 mM MgCl_2_, 2 mM HEPES, 1 mM EGTA, and 0.2% fatty acid–free BSA, pH 7.2 at 37°C) supplemented with 10 mM glutamate and 5 mM malate for complex I–driven respiration in a Seahorse XF96 Extracellular Flux Analyzer. Compounds (4 mM ADP, 2.5 μg/mL oligomycin, 4 μM FCCP, and 2 μM rotenone) were injected to each well in sequence as described previously (30).

### ATP measurement from heart tissue.

ATP production was measured by the ATP assay (MAK190-1KT) according to the manufacturer’s instructions. Briefly, left ventricle tissue was homogenized into ATP assay buffer. Protein concentration of clarified supernatants was determined by BCA assay for further normalization. Clarified supernatants were deproteinized through 10 kDa molecular weight cutoff spin filter (MRCPRT010). Deproteinized homogenate or ATP standards were incubated with ATP probe and converter as standard protocol at room temperature for 30 minutes, protected from light. Fluorescence was measured with an excitation wavelength of 530 nm and fluorescence emission detection at 620 nm.

### Amplex Red–based hydrogen peroxide measurement in heart tissue.

Hydrogen peroxide was measured by the Amplex Red hydrogen peroxide assay (A22188, Thermo Fisher Scientific) according to the manufacturer’s instructions. Briefly, left ventricle was homogenized into Amplex Red reaction buffer. Clarified supernatants or H_2_O_2_ standards were incubated with 100 μM Amplex Red and 0.2 U/mL HRP at room temperature for 30 minutes, protected from light. Fluorescence was measured with an excitation wavelength of 530 nm and fluorescence emission detection at 620 nm. BCA was performed to determine total protein for further normalization.

### Transmission electron microscopy.

Pairs of 6-month-old females and 7-month-old males were sacrificed, and slices (1–2 mm) of left ventricular tissue were fixed in Karnovsky solution (Thermo Fisher Scientific, 50-980-497) overnight at 4°C. Specimens were then washed in PBS before being immersion fixed in 2.5% glutaraldehyde and 2% paraformaldehyde in PBS overnight at 4°C. Fixed samples were washed 3× in PBS, then postfixed in aqueous 1% OsO_4_, 1% K_3_Fe(CN)_6_ for 1 hour. Following 3 PBS washes, the pellet was dehydrated through a graded series of 30%–100% ethanol, 100% propylene oxide, then infiltrated in 1:1 mixture of propylene oxide/Polybed 812 epoxy resin (Polysciences) for 1 hour. After several changes of 100% resin over 24 hours, the samples were embedded in molds, cured at 37°C overnight, followed by additional hardening at 65°C for 2 more days. Semithin (300 nm) sections were heated onto glass slides, stained with 1% toluidine blue, and imaged using light microscopy to ensure proper tissue orientation. Ultrathin (60–70 nm) sections were collected on 100 mesh copper grids, stained with 4% uranyl acetate for 10 minutes, followed by 1% lead citrate for 7 minutes. Sections were imaged using a JEOL JEM 1011 or JEOL JEM 1400 transmission electron microscope (Peabody) at 80 kV fitted with a side mount AMT digital camera (Advanced Microscopy Techniques). The short axis and long axis were calculated according 190 mitochondria from 14 individual images (7 from 6-month female and the other 7 from 7-month male).

### Echocardiography.

Mice (*n* = 8, male, 6 to 7 months old) were anesthetized with 1.5% isoflurane/oxygen, placed on a warming pad, and imaged in the supine position using a Vevo770 ultrasound system with high-frequency 45 MHz RMV707B scan head (VisualSonics, Inc.). M-mode parasternal short-axis scans at papillary muscle level were used to quantify LVEdD and LVEsD from which percentage FS was calculated ([LVEdD − LVEsD]/LVEdD) × 100. Pulse-wave Doppler was used to assess mitral valve flow (E/A ratio) and left ventricular IVRT and MPI, as reliable measures of diastolic function. Electrocardiogram and heart rate recordings were made by attachment of mouse paws to electrodes on the system platform.

### Cell surface area.

AC16 cells (pretreated with control or *hECSIT* siRNA) were cultured in DMEM/12 overnight and treated with angiotensin II (2 μM) for 24 hours. Cells were fixed with 4% (w/v) paraformaldehyde for 15 minutes, permeabilized with 0.1% (v/v) Triton X-100 in PBS for 10 minutes, blocked with a 3% (w/v) BSA solution for 1 hour, and incubated with anti–α-actinin antibody (catalog 05-384, MilliporeSigma) overnight at 4°C. Immunofluorescence was analyzed with a laser scanning confocal microscope, and the surface areas were measured using ImageJ software (NIH). One hundred cells for each condition were randomly measured from 3 individual biological replicates. In some experiments, knockdown cells were allowed to recover for 7 days in DMEM/12 before repeating the procedures above.

### Statistics.

Statistical analysis was performed using an unpaired 2-tailed Student’s *t* test or 1-way ANOVA as specified for multiple comparisons between groups. Data are presented as the mean ± SD unless otherwise indicated. *P* ≤ 0.05 was considered statistically significant.

### Study approval.

All animal experiments were performed in accordance with the regulations and guidelines of the Health Products Regulatory Authority and protocols approved by the Research Ethics committee of Maynooth University. The study utilized a cohort of 38 patients undergoing elective cardiac surgery for coronary artery bypass grafting or valve replacement at the Cardiology Department of the Blackrock Clinic in Dublin, Ireland. Right atrial appendage tissue and peripheral venous blood were collected from this cohort. All subjects gave written informed consent to participate in this study. The Ethics Committee at St Vincent’s University Hospital in Dublin, Ireland, approved all study protocols, which conformed to the principles of the Helsinki Declaration.

## Author contributions

LX developed the concept, designed and performed experiments, analyzed data, prepared the figures, and edited the manuscript; FH developed the concept and designed and performed experiments. ND and BW performed experiments and analyzed data; AH designed, performed and analyzed proteomic studies and performed bioinformatics and immunofluorescence studies; KSE and DJG designed, performed, analyzed, and supervised the echocardiography studies; DBS performed the electron microscopy studies. EO performed the flow cytometry analysis. AMR and RJI performed *S*. *typhimurium* infections. JRH performed the confocal microscopy analysis. NG, CJW, KM, and MTL designed and analyzed the ex vivo clinical study; performed correlation analysis of hECSIT expression and immunohistochemical, biochemical, clinical, and imaging parameters; and edited the manuscript. PNM conceived the study, supervised the overall project, analyzed data, and wrote the manuscript.

## Supplementary Material

Supplemental data

Supplemental Table 3

## Version 1 05/25/2021

In-Press Preview

## Version 2 06/22/2021

Electronic publication
