# Supplementary material for: ECSIT is a critical limiting factor for cardiac function
Source: JCI Insight. 2021 Jun 22;6(12):e142801. doi: 10.1172/jci.insight.142801 (PMC8262467; doi:10.1172/jci.insight.142801)

Supplemental information

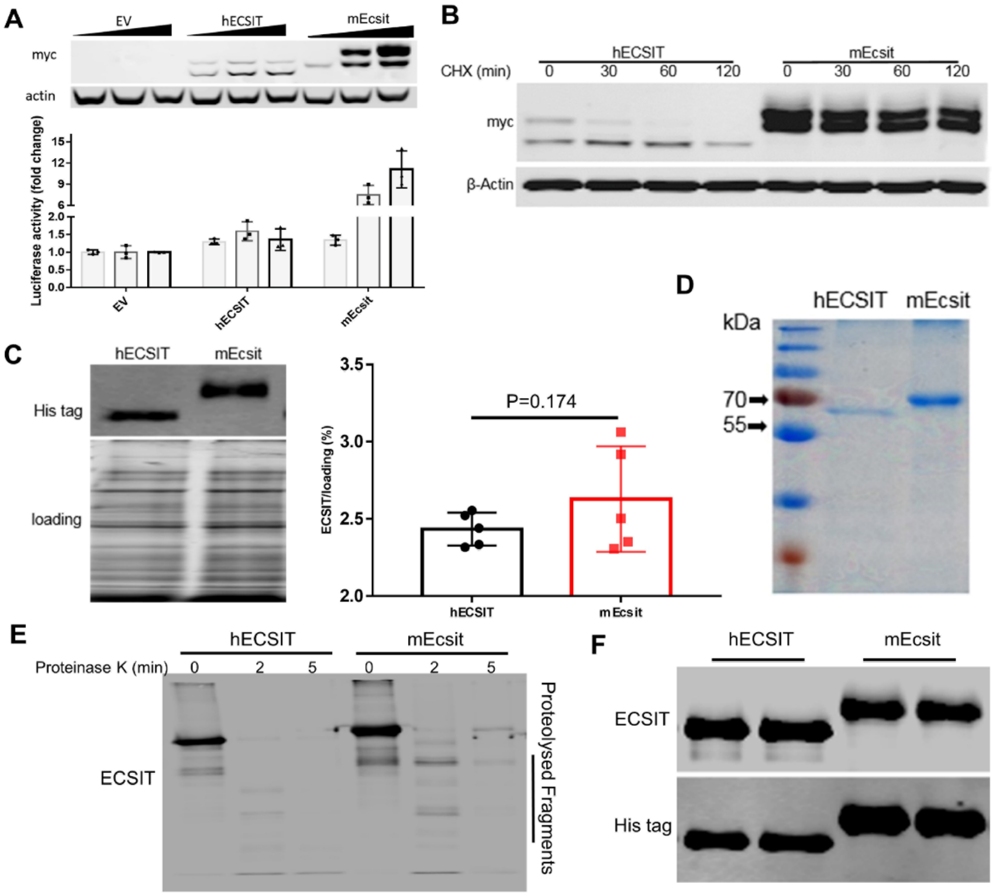

Supplemental Figure 1. Differential Stability of mEcsit and hECSIT. **(A)** Immunoblot analysis, using anti-myc and anti-β–actin antibodies, of cell lysates (upper panels) and fold induction of NFκB-regulated luciferase activity (lower panel) from HEK293T cells transfected with empty vector (EV), myc-tagged hECSIT or myc-tagged mEcsit (hECSIT: 10, 50 and 100 ng; mEcsit: 2, 20 and 100 ng) **(B)** HEK293T cells were transfected with myc-tagged hECSIT or myc-tagged mEcsit and left overnight. Cells were then treated with cycloheximide (100 μM). Cell lysates were generated at indicated times post cycloheximide treatment and subjected to immunoblot analysis, using anti-myc and anti-β–actin antibodies. **(C)** *hECSIT* and *mEcsit* were subcloned into the pET28a-Smt3 vector with in frame tagging of 6×His and Smt3 tags. The plasmid was transformed into *E.coli* Rosetta competent cells and induced by IPTG in 2YT medium. Recombinant hECSIT and mECSIT were detected from *E.coli* lysis using Anti-His. Statistical analysis was from five individual cell culture. (**D**) Recombinant hECSIT and mEcsit yield. The pET28a-Smt3 vector with in frame tagging of 6×His and Smt3 tags was transformed into Rosetta competent cells and induced by IPTG in 2YT medium. Proteins were purified on a Ni affinity column. Protein product was then dialysed into 50 mM Tris buffer (pH 8.0) with 100 mM NaCl. The sizes (kilodalton (kDa)) and mobilities of molecular weight markers are indicated in panels. **(E)** Purified ECSIT proteins (50 μl, 1 μM) were incubated with Proteinase K (4 ng/μl) for indicated times at 37 °C. Reactions were terminated with addition of ice-cold 100% trichloroacetic acid (25 μl) followed by centrifugation at 17,000g for 10 min. The precipitated protein was washed in ice-cold acetone, dried, and resuspended in SDS-PAGE sample buffer. Samples were immunoblotted using an anti-ECSIT antibody. **(F)** Same amount of Recombinant hECSIT and mECSIT (4 μl, 1 μM) was detected by anti-His and anti-ECSIT.

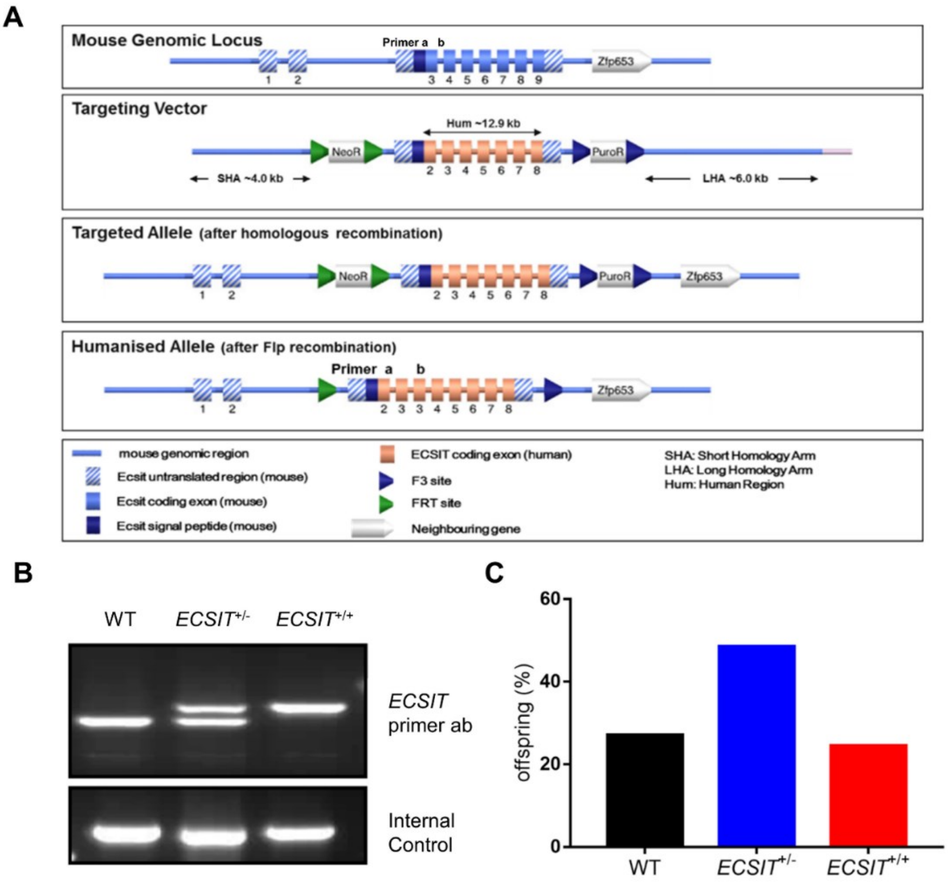

Supplemental Figure 2. Generation of Humanised *ECSIT* mouse. (**A**) Diagram shows the murine *Ecsit* gene; the *Ecsit* targeting vector containing the positive selection Puromycin (PuroR) and Neomycin (NeoR) cassettes ; the targeted Ecsit allele after homologous recombination and the humanised allele after Flp recombination. Homologous recombinants in ES cells were isolated by puromycin and neomycin selection and used for blastocyst injection. Mice that were heterozygous for the targeted allele were bred with mice containing Flp recombinase, under the control of a chimeric CMV enhancer/b-actin promoter, generating constitutive humanized mice in which *Ecsit* exons 3-9 have been replaced with *ECSIT* codons 2-8. Exons are numbered and regions targeted by genotyping primers are also indicated. (**B**) Genotyping by PCR analysis of genomic DNA from ear punches. Primers a and b differentiate the wild type (WT) allele from the *ECSIT* humanised allele in heterozygous (*ECSIT*$^{+/-}$) and homozygous (*ECSIT*$^{+/+}$)) humanised knock-in mice by amplifying a 440 base pair fragment in the WT allele and a 520 base pair fragment in the humanised allele. The integrity of PCR samples was confirmed by using primers to amplify a 585 base pair fragment from the CD79b wild type allele (Internal Control). (**C**) Frequency of wild type, homozygous *ECSIT*$^{+/+}$ and heterozygous *ECSIT*$^{+/-}$ offspring from crossing of heterozygous mice (n=336).

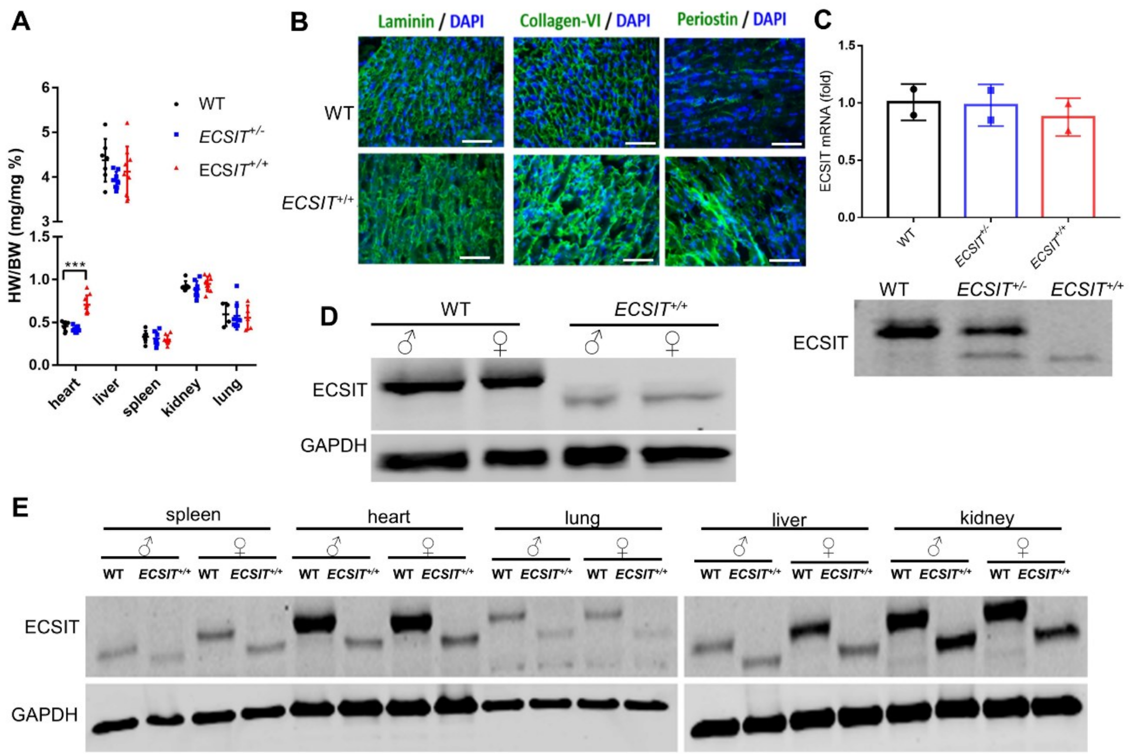

Supplemental Figure 3. Decreased ECSIT level causes cardiac hypertrophy and fibrosis in humanized mice. (**A**) Ratio of tissue weight (TW):body weight (BW) for various tissue in WT and *ECSIT*$^{+/+}$ mice at 7 months. (**B**) Staining of Laminin, Collagen VI and Periostin in left ventricular heart tissue from 6-7 months WT and *ECSIT*$^{+/+}$ mice. Scale bar = 30 µm. (**C**) ECSIT mRNA (upper panel) and protein (lower panel) levels in the left ventricular cardiac tissue of WT, *ECSIT*$^{+/+}$ and heterozygous *ECSIT*$^{+/-}$ mice. (**D**) Western blotting of hECSIT and mEcsit and GAPDH protein in the left ventricular cardiac tissue of 7-month old male and female WT and *ECSIT*$^{+/+}$ mice. (**E**) Western blotting of hECSIT and mEcsit and GAPDH protein in tissue homogenate from spleen, heart, lung, liver and kidney of 7-month old male and female WT and *ECSIT*$^{+/+}$ mice.

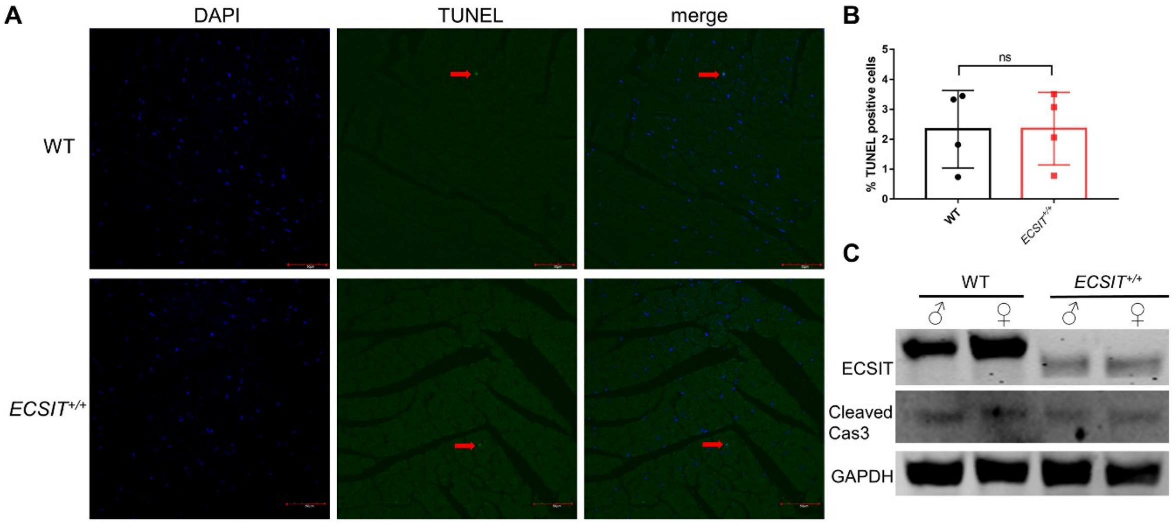

Supplemental Figure 4. Cardiomyocyte apoptosis is not altered in humanized mice. (**A**) TUNEL labelling of heart tissues in WT and *ECSIT*$^{+/+}$ mice at 6-7 months. Arrows indicate spots of TUNEL staining. Scale bar=50 µm. (**B**) Quantification of TUNEL positive cells, n=4. (**C**) Cleaved caspase 3 levels in left ventricular tissue of WT and *ECSIT*$^{+/+}$ mice at 6-7 months. GAPDH is a loading control.

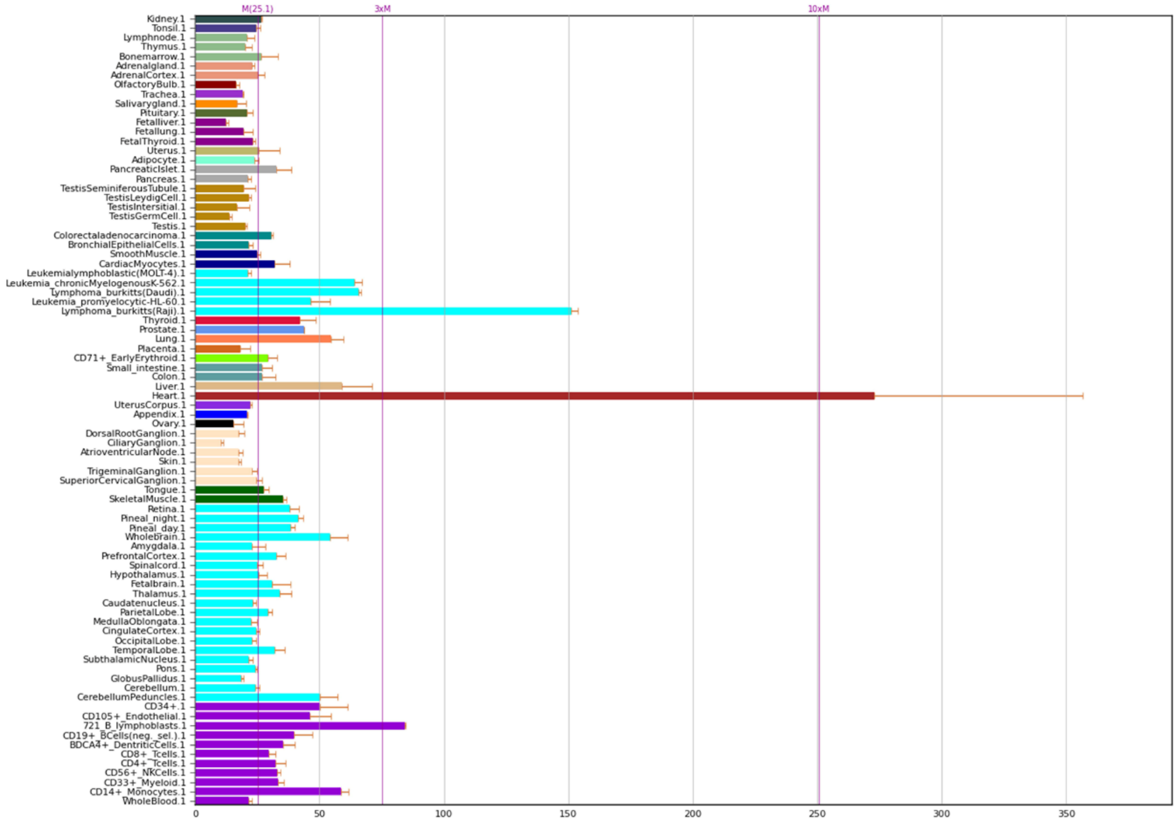

Supplemental Figure 5. hECSIT expression in human tissues and cells.

Relative mRNA expression of *hECSIT* in human tissues and cells as determined using high-density oligonucleotide arrays (http://biogps.org/#goto=genereport&id=51295).

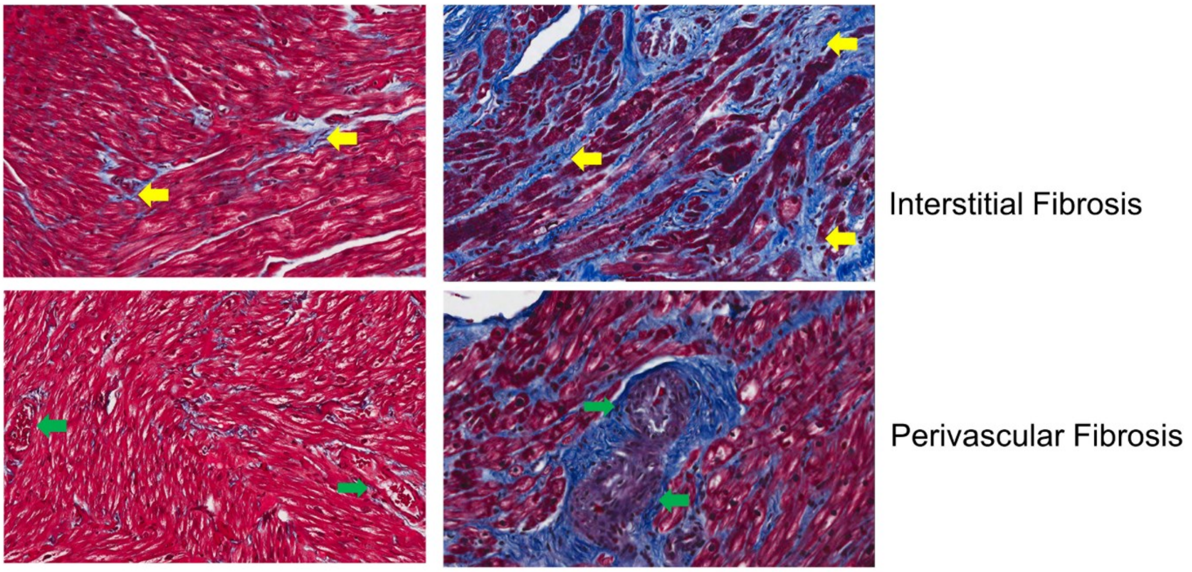

Supplemental Figure 6. Representative images of Masson's Trichrome staining of human heart tissue.

Images represent examples of collagen deposition within the interstitium and perivascular in cardiac tissue stained with Masson's Trichrome. The red staining highlights the cardiac myocytes and the blue staining represents collagen. Yellows arrows highlight interstitial fibrosis and green arrow highlight perivascular regions. Quantification of collagen deposition was achieved using automated positive pixel analysis of digitally scanned slides (Aperio ScanScope XT Slide Scanner) and Imagescope software analysis (Aperio). Digital images captured with a 20x objective.

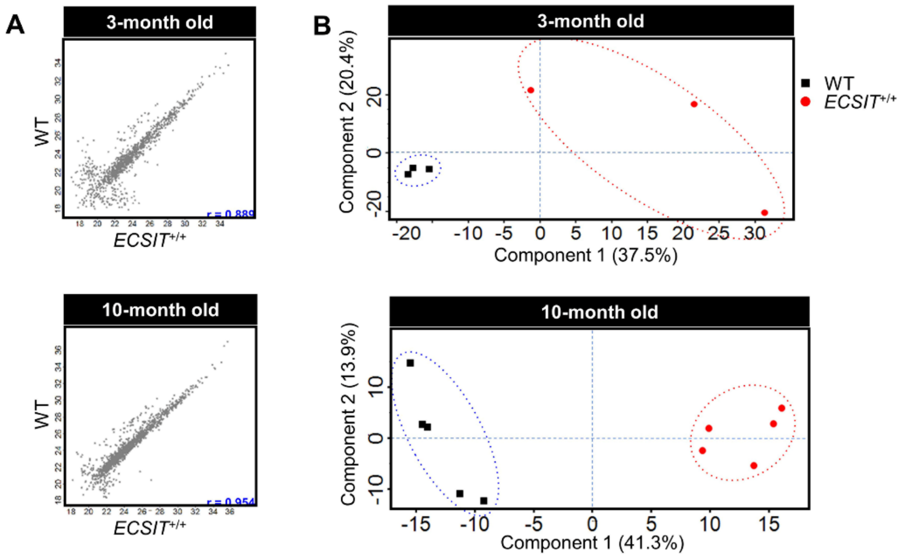

Supplemental Figure 7. MS-based quantitative proteomics illustrates reproducible label-free analysis of 3- and 10- month *ECSIT*$^{+/+}$ cardiac tissue. Heart samples from 3- and 10-month old WT versus *ECSIT*$^{+/+}$ mice were analysed by label-free LC-MS/MS. (**A**) A representative scatter plot of proteomic data for WT versus *ECSIT*$^{+/+}$ of differentially abundant proteins separated by age group was used to illustrate correlation between samples. Pearson correlation (r) was used to demonstrate significant correlation between samples. (**B**) Principal component analysis (PCA) was used to investigate underlying differences between conditions from quantitative proteomic data. WT and *ECSIT*$^{+/+}$ features, for both 3- and 10-month cluster together.

Supplemental Table 1. Patient characteristics as described by clinical demographic and doppler echocardiographic criteria

	Total Population (n=38)	Left Ventricular Hypertrophy (n=16)	Controls (n=22)	
**Demographics**	Mean (SD) / N (%)	Mean (SD) / N (%)	Mean (SD) / N (%)	*p*
Age (years)	68.0 (9.6)	65.1 (9.5)	70.1 (9.8)	-
Male	28 (74%)	8 (50%)	20 (91%)	<0.01
**Cardiovascular History**				
Ischemic Heart Disease	27 (71%)	11 (69%)	16 (73%)	-
Valvular Disease	18 (47%)	7 (44%)	11 (50%)	-
Atrial Fibrilation	9 (24%)	1 (6.0%)	8 (36%)	-
Diabetes Mellitus	7 (18%)	2 (13%)	5 (23%)	-
Hypertension	24 (63%)	8 (50%)	16 (72%)	-
Dyslipidaemia	23 (61%)	8 (50%)	15 (68%)	-
**Medications**				
Any RAAS Modifying Therapy	17 (45%)	6 (38%)	11 (50%)	-
Beta Blocker	24 (63%)	11 (69%)	13 (59%)	-
Diuretic	14 (37%)	5 (31%)	9 (41%)	-
Calcium Channel Blocker	4 (10%)	1 (6%)	3 (14%)	-
Statin	22 (58%)	7 (44%)	15 (68%)	-
**Cardio-Metabolic Phenotype**				
Body Mass Index (Kg/m^2)	27.1 (3.2)	27.1 (3.8)	27.1 (2.8)	-
Systolic Blood Pressure (mmHg)	135 (7)	133 (7.5)	137 (7)	-
Diastolic Blood Pressure (mmHg)	80 (7)	78 (7)	81 (7)	-
Creatinine (mmol/L)	92 (14)	94 (15)	92 (14)	-
**Doppler Echocardiography**				
EF (%)	56 (8.0)	55 (10)	57 (6)	-
E/E'	9.4 (2.7)	9.9 (3.4)	9.1 (2.0)	-
LAVI (ml/m^2)	28.6 (3.9)	28.0 (2.6)	29.0 (4.6)	-
LVMI (g/m^2)	111 (24)	129 (22)	98 (15)	<0.0001
**Immunohistochemical Analyses**				
Collagen Volume Fraction (Masons Trichrome Stain)	46.3 (17.7)	44.7 (11.8)	47.5 (21.1)	-
Collagen Volume Fraction (Picosirus Red %)	49.4 (8.8)	49.2 (8.8)	49.6 (9.0)	-
ECSIT Lavg	117.7 (14.5)	112.5 (10.7)	121.6 (15.9)	<0.05

Abbreviations: EF, Ejection Fraction; E/E', tissue Doppler early diastolic mitral annular velocity; LAVI, Left Atrial Volume Index; LVMI, Left Ventricular Mass Index.

Supplemental Table 2. Echocardiogram and ECG analysis of 6-7-month WT, *$ECSIT^{+/-}$* and $ECSIT^{+/+}$ mice

	WT	*$ECSIT^{+/-}$*	*$ECSIT^{+/-}$*
LVPWS (mm)	0.72±0.083	0.71±0.06	1.10±0.06**
LVPWD (mm)	0.92±0.08	1.03±0.05	1.24±0.07*
E/A Ratio	1.63±0.09	1.85±0.11	1.51±0.04
IVRT (ms)	16.8±0.69	13.8±0.52	25.5±3.29**
MPI	0.62±0.04	0.50±0.02	0.80±0.05**
Heart Weight (g)	0.17±0.01	0.17±0.02	0.26±0.01**
P-R Interval (ms)	47.2±1.29	44.9±1.77	59.6±2.81***
P-wave Duration (ms)	9.88±0.414	9.69±0.540	10.96±0.50
QRS Complex Duration (ms)	11.45±0.2648	10.87±0.17	13.38±0.607**
Q-T Interval (ms)	55.84±3.15	55.32±1.77	63.47±1.70
T-wave Duration (ms)	42.39±3.14	42.81±1.82	48.13±2.21

Abbreviations: LVPWS, Left ventricular posterior wall end systole; LVPWD, Left ventricular posterior wall end diastole; E/A, Peak velocity flow in early diastole (E wave) / peak velocity flow in late diastole caused by atrial contraction; IVRT, Isovolumic relaxation time; MPI, Myocardial perfusion imaging.

*p<0.05, **p<0.01, ***p<0.001 relative to WT

Supplemental Table 3. List of Differentially Expressed Proteins in WT and *ECSIT*$^{+/+}$ heart tissue

Supplemental Table 4. Key resource table

REAGENT or RESOURCE	SOURCE	IDENTIFIER	Dilution
Antibodies			
α-actinin	Merck Millipore	05-384	1:100
COX IV	CST	4844	1:1000
COX V	Invitrogen	A-21351	1:2000
DRP1	CST	14647	1:1000
p-DRP1	CST	3455	1:1000
ECSIT	Novus	NBP1-91858	1:1000
Fis1	GeneTex	GTx111010	1:2000
BNIP3L	Thermo Fisher	PA5-29385	1:1500
FUNDC1	Novus	NBP1-81063	1:500
His tag	Biolegend	652502	1:3000
MFN1	Novus	NBP1-51841	1:500
MFN2	CST	D2D10	1:1000
Myc tag	CST	2276	1:1000
NUDFS3	SANTA CRUZ	sc-374282	1:500
OPA1	Novus	NB110-55290	1:500
p62	CST	5114	1:1000
Parkin	CST	4211	1:1000
PINK1	Novus	BC100-494	1:1000
Tom 20	CST	D8T4N	1:1000
IκBα	CST	4814	1:1000
p-IκBα	CST	9246	1:1000
JNK	CST	9252	1:1000
p-JNK	CST	9251	1:1000
ERK	CST	9102	1:1000
p-ERK	CST	9101	1:1000
p38	CST	8690	1:1000
p-p38	CST	4511	1:1000
Cleaved caspase3	CST	9664	1:1000
GAPDH	CST	2118S	1:1000
β-actin	Sigma	AC-15; A1978	1:5000
mouse IRDyeTM 680	LI-COR	926-68070	1:5000
rabbit IRDyeTM 800	LI-COR	926-32211	1:5000
Laminin	Novus	NB300-144	1:100
Collagen-VI	Novus	NB120-6588	1:200
Periostin	abcam	ab14041	1:500
Goat anti-Rabbit IgG (H+L) Highly Cross-Adsorbed Secondary Antibody, Alexa Fluor Plus 594	Thermo Fisher	A32740	1:200
Goat anti-Mouse IgG (H+L) Highly Cross-Adsorbed Secondary Antibody, Alexa Fluor Plus 488	Thermo Fisher	A32723	1:200
Bacterial and Virus Strains			
Top10	Invitrogen	C4040-03	
Rosetta (DE3)	Novagen	70954-3	
Biological Samples			

Right atrial appendage tissue and peripheral venous blood	Cardiology Department of the Blackrock Clinic in Dublin	N/A	
Chemicals, Peptides, and Recombinant Proteins			
FCCP	Sigma	C2920-10MG	
oligomycin	Sigma	O4876-5MG	
2-Deoxy-D-glucose	Sigma	D8375-5G	
L-Glutamine Soultion (100X)	Merck Millipore	TMS-002-C	
Antimycin A	Sigma	A8674-25MG	
Rotenone	Sigma	R8875-1G	
ADP	Sigma	A2754	
D-Mannitol	Sigma	M9546	
EGTA	Sigma	E3889	
Glutamate	Sigma	G8415	
Malate	Sigma	M6413	
Sodium succinate dibasic hexahydrate	Sigma	S2378	
4% formaldehyde	Thermo Fisher	R37814	
Xylene	Sigma	534056	
Wheat Germ Agglutinin	Invitrogen	W834	
Lipofectamine 2000	Invitrogen	11668-019	
Urea	Sigma	U5378-500G	
Imidazole	Sigma	56750-100G	
IPTG	Sigma	I6758-1G	
Lys-C	Promega	V1071	
Trypsin	Promega	V5111	
ProteaseMAX™ Surfactant, Trypsin Enhancer	Promega	V2071	
Recombinant hECSIT	This paper	N/A	
Recombinant mEcsit	This paper	N/A	
Sodium pyruvate solution	Sigma	S8636-100ML	
NADH	Sigma	10128023001	
Nitrotetrazolium Blue chloride	Sigma	N6876-250MG	
3,3′-Diaminobenzidine	Sigma	D8001-1G	
Cytochrome c	Sigma	C2506-50MG	
Angiotensin II human	Sigma	A9525-10MG	
MitoSOX	Invitrogen	M36008	
CM-H2DCFDA	Invitrogen	C6827	
Critical Commercial Assays			
Seahorse XF Cell Mito Stress Test Kit	Agilent	103015-100	
Seahorse XF Glycolysis Stress Test Kit	Agilent	103020-100	
Amplex Red Hydrogen Peroxide assay Kit	Thermo Fisher	A22188	
ATP assay Kit	Sigma	MAK190-1KT	
TUNEL staining Kit	Promega	G3250	
H&E staining Kit	abcom	Ab245880	
Trichrome Stain Kit	Sigma	HT15-1KT	
qScript™ cDNA Synthesis Kit	VWR	733-1175	
Pierce™ BCA Protein Assay Kit	Thermo Fisher	23227	

Dual-Luciferase Reporter Assay System	Promega	E1910	
Lipofectamine™ RNAiMAX Transfection Reagent	Invitrogen	13778030	
NativePAGE™ Running Buffer Kit	Thermo Fisher	BN2007	
NativePAGE™ Sample Prep Kit	Thermo Fisher	BN2008	
SYBR Green SuperMixes and FastMixes™ with ROX reference dye	VWR	733-1386	
KARNOVSKY'S FIXATIVE KIT	Thermo Fisher	50-980-497	
L-lactate assay Kit	Sigma	MAK064-1KT	
Vectastain ABC kit	Vector Laboratories	PK-6100	
Experimental Models: Cell Lines			
HEK293T	ATCC	CRL-11268	
AC16	Merck Millipore	SCC109	
Experimental Models: Organisms/Strains			
Mouse: C57BL/6N-WT	This study	Inbreed from C57BL/6N-ECSIT+/-	
Mouse: C57BL/6N-ECSIT+/-	TaconicArtenis	Generated from TaconicArtemis C57BL/6N Tac ES cell	
Mouse: C57BL/6N-ECSIT+/+	This study	Inbreed from C57BL/6N-ECSIT+/-	
Oligonucleotides			
Silencer™ Select Negative Control No. 1 siRNA	Thermo Fisher	4390844	
human ECSTI siRNA	Thermo Fisher	4390825 (Assay ID s224197)	
Mice genotyping primer-F: CCATTTGCTGGAGTCTGTTCC	Integrated DNA Technologies	N/A	
Mice genotyping primer-R: AAATTCAGCACCTACATGGCAG	Integrated DNA Technologies	N/A	
ECSIT-qPCR1-F : CAGCGCATCTTCGTCCACTAC	Sigma	N/A	
ECSIT-qPCR1-R : AACTTGAGCATGGGGTA	Sigma	N/A	
mtCOI-F : TGCTAGCCGCAGGCATTAC	Sigma	N/A	
mtCOI-R : GGGTGCCCAAAGAATCAGAAC	Sigma	N/A	
Ndufv1-F : CTTCCCCACTGGCCTCAAG	Sigma	N/A	
Ndufv1-R : CCAAAACCCAGTGATCCAGC	Sigma	N/A	
PCPE-F: CCAACTACACCAGACCCGT	Sigma	N/A	
PCPE-R: CGGAATGAGAGGGACACAGT	Sigma	N/A	
PCP-F: TCTCCATCGGCAAGAACTGT	Sigma	N/A	

PCP-R: ATGCCCCTGGAGAATGTGTT	Sigma	N/A	
LOX-F: GTCTGGCCAGTACAGCATACAG	Sigma	N/A	
LOX-F: TTGGCATCAAGCAGGTCATA	Sigma	N/A	
B2M-F: AGGCTATCCAGCGTACTCCA	Sigma	N/A	
B2M-R: CCAGTCCTTGCTGAAAGACA	Sigma	N/A	
HPRT-F: GTCCCAGCGTCGTGATTAGC	Sigma	N/A	
HPRT-R: TGGCCTCCCATCTCCTTCA	Sigma	N/A	
Recombinant DNA			
pcDNA3.1	Invitrogen	V79020	
ECSIT/mutants in pcDNA3.1	This paper	N/A	
NFκB-luciferase	Agilent	219077	
pRL Renilla-TK	Promega	E2241	
Software and Algorithms			
ImageJ	NIH	https://imagej.nih.gov/ij/	
Xcalibur	Thermo Fisher	OPTON-30487	
MaxQuant	N/A	https://www.maxquant.org/maxquant/	
Perseus	N/A	https://www.maxquant.org/perseus/	
STRING	N/A	https://string-db.org/	
PANTHER	N/A	http://pantherdb.org/	
Other			
Seahorse XF DMEM Medium, pH 7.4	Agilent	103575-100	
Dulbecco's Modified Eagle's Medium	Fisher	10103542	
Dulbecco's Modified Eagle's Medium/Nutrient Mixture F-12 Ham	Sigma	D6434-6X500ML	
Ni Sepharose 6 Fast Flow	GE Healthcare	17-5318-01	
NativePAGE™ 3-12% Bis-Tris Protein Gels	Thermo Fisher	BN1001BOX	
C18 spin columns	Fisher	89870	
Chamber slide system	Fisher	154534PK	
2xYT Medium EZMix	Sigma	Y2627-1KG	

Methods (supplementary)

Cell Culture

For isolation of BMDMs, tibias and femurs were removed from WT, *ECSIT* $^{+/-}$ and *ECSIT* $^{+/+}$ mice by sterile techniques and bone marrow was flushed with fresh RPMI-1640 plus GlutaMAX-I medium using a 271/4 gage needle. Cells were plated in medium supplemented with 10% (v/v) conditioned medium of L929 mouse fibroblasts and were maintained for 6 days at 37°C in a humidified atmosphere of 5% CO2. HEK293T cells were cultured in Dulbecco's modified Eagle's medium supplemented with 10% (v/v) fetal bovine serum, 50U/ml penicillin and 50µg/ml streptomycin. AC16 cells were cultured in DMEM/F12 (Sigma, D6434) containing 2 mM L-Glutamine, 12.5% FBS and 50U/ml penicillin and 50µg/ml streptomycin. All cell lines were cultured at 37°C in a humidified atmosphere of 5% CO2. Medium was replaced every 2-3 days.

Transient transfection

HEK293T cells were seeded at $5x10^5$ cells/well in 6-well plates and left overnight or until approximately 70% confluence. 1 µg of plasmid DNA as indicated was diluted in 250 µl of Opti-MEM and mixed gently. In addition, 4 µl of Lipofectamine 2000 was diluted into 250 µl of Opti-MEM. After 5 min incubation at RT, the solutions were combined together and incubated for 20 min at RT. 500 µl of the DNA-Lipofectamine complexes were added to each well and left for 24 h before harvesting.

NFκB Luciferase assay

HEK293T cells were seeded at $4 x10^4$/well in 96-well plate and grown for 24h. NFκB regulated firefly luciferase reporter plasmid (80 ng), constitutively expressed *Renilla*-luciferase reporter construct pRL Renilla-TK (40 ng) and varying amounts of ECSIT expression constructs (0-100 ng) were transfection with 0.5 µl Lipofectamine 2000. Luminescence was detected by the Dual luciferase assay (Promega) according to the manufacturer's instructions.

Immunoblotting

For whole cell lysate analysis cells were lysed in 1×SDS-PAGE loading buffer. For tissue lysate analysis, tissues were homogenised in RIPA buffer (50 mM HEPES pH 7.5, 10% (v/v) glycerol, 0.5% (w/v) CHAPS, 0.5% (v/v) Triton-X-100, 150 mM NaCl, 1 mM Na3VO4, 1 mM EDTA, 1 mM PMSF and complete protease inhibitor mixture). Concentration was confirmed by BCA assay and samples subjected to 10-20% SDS-PAGE. Protein was transferred to nitrocellulose membranes and analysed by immunoblot with according antibodies.

Immunoreactivity was visualized by the Odyssey Imaging System (LICOR Biosciences) or enhanced chemiluminescence.

Quantitative RT-PCR

Total RNA was isolated from mouse ventricular myocardium with TRIzol reagent (Invitrogen) according to the manufacturer's instructions, and then converted to cDNA using cDNA Synthesis Kit (733-1175). Quantitative RT-PCR reactions were performed using SYBR Green SuperMixes (733-1386) and a StepOnePlus Real-Time PCR System. The primers used see **S4 Table**.

Salmonella typhimurium Infection

BMDMs were plated at 2x10^6 cells in 6-well plates or 2x10^5 in 96-well plates in BMDM medium with 10% FBS without antibiotics. After overnight rest, cells were infected with *S. Typhimurium* (SL1344) at the indicated time points and multiplicities of infection. Bacteria were grown from single colonies in LB broth at 37ºC overnight and following overnight incubation, bacteria were refreshed and incubated at 37ºC with continuous gentle agitation for 2h. Bacteria were subsequently collected, washed in endotoxin free PBS three times and Optical density (OD; 600) was measured and adjusted accordingly. *S. Typhimurium* (SL1344) was a kind gift from Prof. M Valvano (Queen's University Belfast, Belfast, United Kingdom). Cells were infected for 2 h and following this, the media was removed, cells were washed with PBS and media was replaced with DMEM containing gentamicin (50 ug/ml) to kill extracellular bacteria.

For *in vivo* studies, 1 ml of overnight culture of *S. typhimurium* (strain SL1334) was diluted in 19 ml of nutrient media (Sigma Aldrich), supplemented with 50 μg/ml streptomycin. The culture was incubated at 37°C with continuous gentle agitation for 2 h. The bacteria were then pelleted by centrifugation at 5000 G and washed 3 times with sterile endotoxin free PBS. The Optical density (OD; 600 nm) was measured and adjusted to an OD of 0.5. Female WT, *ECSIT*$^{+/-}$ and *ECSIT*$^{+/+}$ litter mates were inoculated orally with 100 μl of bacteria (1 x 10^7 CFU) suspended in sterile saline or sham inoculated with injection grade saline using a flexible disposable feeding needle (Thermo Fischer Scientific). All animals were carefully monitored for weight loss and symptoms of systemic infection. At 72 h post-inoculation, the animals were sacrificed by administration of a terminal dose of anaesthetic (Ketamine/xlyazine). All tissue samples were weighed and then homogenised using a handheld electric homogeniser, and serial dilutions plated on nutrient agar with 50 μg/ml streptomycin. After overnight incubation at 37ºC, the colonies were counted and the CFU/g of tissue calculated.

ROS measurements by flow cytometry

BMDMs were infected with *Salmonella* (MOI 10) for 2 h. Culture medium was removed, cells were washed with PBS, then incubated with MitoSOX (to measure the mROS superoxide) or CM-H2DCFDA (to measure total cellular H_2O_2) (Invitrogen) at 2.5 mM final concentration in serum-free DMEM (Invitrogen) for 30 min at 37 °C. Cells were washed with warmed PBS, removed from plates with cold PBS containing 1 mM EDTA by pipetting. Cells were pelleted at 500 g for 5 min, and then re-suspended in cold PBS containing 1% FBS. Samples were acquired using an Attune NxT flow cytometer (ThermoFisher Scientific) and analysed using FlowJo software.Unstained controls were treated similarly, except that dyes were omitted. To control for baseline dye fluorescence, samples from each experiment were left unstimulated but stained according to the above procedure.

Sample preparation for MS analysis

Cardiac tissue was homogenised in the presence of a lysis buffer (7 M urea, 2 M thiourea, 65mM CHAPS, 100 mM DTT, supplemented with protease and phosphatase inhibitors) with an IKA Ultra Turbax blender and incubated for 2.5 h at 4°C. The crude extract was clarified by centrifugation at 16,000 g for 20 minutes. Protein samples were processed using a ReadyPrep 2-D clean up kit and resuspended in 6 M urea, 2 M thiourea (in 10 mM Tris-HCl, pH 8.0). Protein concentration was determined using the Bradford method. Tissue lysates containing 25 µg protein was prepared for label-free mass spectrometry analysis. Protein lysates were reduced with 10mM DTT for 30 min at 37°C and alkylated with 55 mM iodoacetamide for 30 min at room temperature in the dark. Samples were digested with 0.5 µg Lys-C for 4 h at 37°C. Samples were subsequently diluted with four volumes 50 mM ammonium bicarbonate and further digested with 1 µg trypsin and incubated at 37°C overnight. The following day, digestion was terminated by acidification with 2% trifluoroacetic acid (TFA) in 20% acetonitrile (3:1 (v/v) dilution). Peptides were purified using C18 spin columns, lyophilised and stored at -20°C until MS analysis.

Liquid chromatography-mass spectrometry analysis

MS analysis was performed using a Q-Exactive mass spectrometer (Thermo Fisher Scientific) (coupled on-line to an Ultimate 3000 NanoHPLC system (Dionex Corporation, Sunnyvale, CA, USA) via a nano electrospray source. Lyophilised cardiac peptides were resolved in 0.1% formic acid and 1µg of peptides was used for MS analysis and loaded onto a C18 trap column (C18 PepMap, 300µm id × 5 mm, 5µm particle size, 100 Å pore size; Thermo Fisher Scientific). The column temperature was set to 45°C. The trap column was switched on-line with an analytical Easy Spray column (C18 PepMap, 75µm id × 500 mm, 2µm particle size, 100 Å

pore size; Dionex). Peptides were eluted with solvent A (0.1% (v/v) formic acid and 80% (v/v) acetonitrile) and eluted over a 180 min gradient from 5%-45% solvent A for 120 min, 45% solvent A for 2.5 min, 90% solvent A for 9 min and 3% solvent A for 43 min, at a flow rate of 300 nL/min. Data was acquired using Xcalibur software (Thermo Scientific). The mass spectrometer was operated in data-dependent and positive mode and was externally calibrated. MS survey scans (120 to 1800 *m/z*) were acquired with a resolution of 140,000, and lock mass set to 445.12003. A top-15 method was used to select up to the fifteen most abundant precursor ions with a charge of ≥ 2. Selected precursor ions were subjected to high-energy CID (collision-induced dissociation) fragmentation, and a resolution of 17,500. An isolation window of 2 *m/*z and one microscan were set to collect tandem mass spectra and dynamic exclusion window of sequenced peptides was set to 40 s.

MS data analysis

MS raw files were processed with MaxQuant software, version 1.5.0.0. The raw files were searched against the mouse, *Mus Musculus,* UniProt KB FASTA database (16,844 proteins, released August 2016) for MS/MS based peptide identification via Andromeda. Enzyme specificity was set to Lys-C and trypsin, with a minimum number of seven amino acids required for peptide identification, a maximum of two missed cleavages and the FDR was set to <1% at the peptide and protein level. Default settings were used for variable and fixed modifications (variable modification; acetylation (N-terminus) and methionine oxidation; fixed modifications; carbamidomethylation). A target-decoy approach to identify peptides and proteins at an FDR <1% (Cox *et al.,* 2011). Peptide identification was performed with the precursor mass deviation up to 4.5 ppm after time-dependent mass calibration and an allowed fragment mass deviation of 20 ppm. For label-free protein quantification, the MaxLFQ algorithm was employed for intensity determination and normalisation procedures and is fully compatible with peptide and protein separation prior to MS analysis. The minimum ratio count was set to two. Quantitation of high-resolution peptide profiles was based on mass-to-charge (*m/z*), retention time and intensity values. MaxQuant was used to calculate pairwise protein ratios by taking the median of all pairwise peptide ratios per protein. Only shared identical peptides were considered for each pairwise comparison, with a minimum number of one ratio count required for each pairwise comparison and a least-squares analysis was employed to rebuild the relative abundance profile for individual proteins. This facilitated the conservation of the total summed intensity for a protein across all the samples under analysis. 'Match between runs' was enabled for sample processing to maximise the number of quantification

events across samples, permitting the quantification of high-resolution MS1 features that were not identified by MS2 in each single measurement. For matching between runs, the retention time alignment window was set to 30 sec and the match time window was 1 min. For data handling, normalisation, statistics and annotation enrichment analysis freely available open-source bioinformatics platforms Perseus (version 1.5.5.3) or R-studio (version 0.99.903) (R Development Core team, 2011) were employed. Cardiac protein analysis of LFQ data was initially filtered by excluding proteins that were identified only by site modification, contaminants, found in the decoy database or quantified in only one of the replicates. Sample LFQ intensity values were log2 transformed and samples were assigned to their corresponding groups. Missing values were imputed on the basis of a normal distribution (width = 0.3, downshift = 1.8) to replace missing values with numbers that represent low abundant proteins. For pairwise comparison of proteomes, a two-side *t*-test statistic was employed. All other statistical and bioinformatics data analyses were performed using the freely available software STRING (version 10.0) and PANTHER, version 11.1. STRING (http:string-db.org/) was used to illustrate known and predicted protein-protein interactions. Data was displayed in confidence view using the high confidence (0.700) parameter for analysis. Differentially expressed protein accession numbers were submitted to PANTHER (http://pantherdb.org/) to perform GO analysis.

Immunofluorescence

Mice (6-7 months) were sacrificed and hearts dissected and fixed in 4% (v/v) paraformaldehyde for 2 h. Tissue was then processed for immunohistochemistry. Briefly, 6 μm-thick heart cryosections were incubated overnight at 4°C with primary antibodies, diluted in 10% foetal calf serum and 0.5% Triton X-100 in PBS. Heart sections were then incubated with appropriate fluorophore-conjugated secondary antibodies for 2 h at room temperature. Cover-slipped were applied to sections with Vectashield-DAPI (Vector Labs, Burlingame, CA) and examined by confocal microscopy (C1 Nikon Eclipse TE200-U, Nikon UK Ltd, Surrey, UK). For wheat germ agglutinin (WGA) staining, 5 μg/ml WGA was incubated with fixed slides for at least 2h.

Confocal morphometry

Confocal images were used to quantify the expression (as measured by the mean fluorescence intensity - MFI) of ECSIT in heart sections (n=3 animals per group; 2 sections per animal; 4 images per heart location). During image acquisition, confocal settings remained constant and images were then analyzed using FIJI software (National Institutes of Health, Bethesda, MD). Images were obtained using a 63x water immersion objective (Olympus Corporation, Tokyo,

Japan) in myocardial regions lining the left ventricle. MFI values were estimated from manually traced areas across the above heart locations, from an average selection of at least 1000 pixels to ensure a high degree of variance. Background images were acquired from a vacant area of the labelled section and subtracted from the raw images to eliminate background noise. At least 24 heart images per heart/mouse strain were analysed and used for statistical purposes. MFI values at heart locations were compared between different strain of mice by one-way analysis of variance (ANOVA), followed by post-hoc Bonferroni's pairwise comparisons. Data were expressed as mean ± SEM and $p<0.05$ was considered statistically significant.

Histology

At post-mortem, samples of cardiac tissue were fixed in neutral buffered formalin. Following fixation tissues were paraffin-embedded, sectioned at 5µm and stained with haematoxylin and eosin (H&E). The H&E staining Kit (Ab245880) was used according to the manufacturer's specifications. Fibrosis of mice hearts was detected using Trichrome stain kit (HT15-1KT) according to the manufacturer's specifications. Staining was viewed with an E-800 Eclipse microscope (Nikon). The fibrosis areas were measured using ImageJ software.

TUNEL staining

Heart tissue were fixed in neutral buffered formalin and were paraffin-embedded, sectioned at 5µm. After deparaffinization in xylene, slides were rehydrated through graded ethanol washes (100%, 95%, 85%, 70%, 50%). Pre-equilibration and rTdT reaction were performed according to the manufacturer's specifications. Staining was viewed with a confocal microscope.

Expression and purification of recombinant ECSIT proteins

cDNA encoding hECSIT and mEcsit were subcloned into the pET28a-Smt3 vector in frame with 6×His and Smt3 tags. Plasmids were transformed into Rosetta competent cells and induced by IPTG (0.5 mM) in 2YT medium for 16-24 h at 16 °C . Proteins were purified on a Nickel (Ni) affinity column (GE Healthcare) in 50 mM Tris buffer (pH 7.5) containing 300 mM NaCl and 3 mM β-mercaptoethanol. Sonicated cell lysates were loaded into the Ni affinity column, then washed by the same buffer containing 50 mM imidazole. Finally proteins were eluted by the same buffer containing 200 mM imidazole. Protein product was then dialysed into 50 mM Tris buffer (pH 8.0) containing 100 mM NaCl.

Protease K digestion assay
ECSIT proteins (50 µl, 1 µM) were incubated at 37 °C with Proteinase K (4 ng/µl) for 0-5 min. Reactions were terminated by addition of ice-cold 100% trichloroacetic acid (25 µl) and the mixture was centrifuged at 17,000 g for 10 min. The supernatant was carefully removed, and precipitated protein washed in ice-cold acetone, dried, and resuspended in 50 µl SDS-PAGE sample buffer. The protein products were analyzed by western blot using an anti-ECSIT antibody.

Patient samples analysis

Tissue preparation
Atrial biopsies were collected at onset of surgery and immediately divided into two parts and either stored in Allprotect Tissue Stabilization Reagent (Qiagen) for subsequent RNA extraction, or were formalin-fixed for histological analysis.

Peripheral blood sampling
Peripheral venous blood were obtained at the time of clinical assessment/surgery. Serum samples were obtained following centrifugation at 2500 g for 10 min at 4ºC. Samples were aliquoted and stored at –80ºC until required. Each serum sample underwent no more than three freeze–thaw cycles prior to its use in enzyme-linked immunosorbent assays (ELISA) and radio-immuno assays (RIA).

Picrosirius red tissue staining and image analysis
Tissue sections (5 µm thick) were rehydrated and incubated with 0.2% phosphomolybdic acid for 2 minutes. Following a rinse in distilled water, the slides were stained with picrosirius red (Direct Red 80 in picric acid, Sigma) for 90 minutes. The slides were then placed in 0.4% HCl for 2 minutes, 70% ethanol for 45 seconds, dehydrated, and cover-slipped for analysis.

The degree of collagen deposition was quantified by automated digital image analysis (Aperio ScanScope XT Slide Scanner, Aperio Technologies) at 20-fold magnification. Automated image analysis was performed using ImageScope (Aperio, Wetzlar, Germany). A positive pixel count algorithm was used to quantify dark pink-stained collagen within each image. Required analysis input parameters for each stain were based on the hue, saturation, and intensity colour model. To detect collagen with picrosirius red, a hue value of 0.8 was specified, and a hue width of 0.5 was used to include the moderate range of colour shades, as previously described PMID: 26130616.

Masons trichrome tissue staining and image analysis

Masson's trichrome (MTC) staining kit (Dako) was used for analysis of interstitial collagen within the myocardial tissue. Tissue sections (8 μm) were rehydrated and incubated overnight in Bouin's solution. Sections were then incubated with Weigert's hematoxylin, Biebrich Scarlet-acid Fuchsin solution, phosphotungstic/phosphomolybdic acid solution and Aniline Blue solution. Following this, tissue sections were incubated in acetic acid and subsequently dehydrated and cover-slipped for analysis. Image analysis of MTC staining was performed by automated analysis using the Aperio ScanScopeXTSlide Scanner system at 20-fold magnification, and ImageScope software, as previously described PMID: 24301681. A positive pixel count algorithm was used to quantify blue-coloured collagen within each scanned image. A hue value of 0.66 was specified. The default hue width value of 0.5 was used to allow inclusion of a moderate range of colour shades. A collagen volume fraction was calculated based on the percent of blue collagen staining quantified within a tissue section.

Tissue gene expression analysis

For analysis of myocardial tissue gene expression, the tissue was individually disrupted and homogenized using an Ultra Turrax T25 Dispersing Instrument (IKA). RNA was extracted using the AllPrep DNA/RNA extraction kit (Qiagen) according to the manufacturer's instructions. First strand cDNA synthesis was carried out using SuperScript II RT (Invitrogen). Quantitative real-time polymerase chain reaction (QPCR) primers were designed for Procollagen C-proteinase Enhancer (PCPE), Procollagen C-proteinase (PCP), and Lysyl Oxidase (LOX). Primer sequences used were are follows: PCPE: CCAACTACACCAGACCCGT (F), CGGAATGAGAGGGACACAGT (R); PCP: TCTCCATCGGCAAGAACTGT (F), ATGCCCCTGGAGAATGTGTT (R); LOX: GTCTGGCCAGTACAGCATACAG (F), TTGGCATCAAGCAGGTCATA (R). QPCR reactions were normalized to the housekeeping gene beta-2-microglobulin (B2M): AGGCTATCCAGCGTACTCCA (F), CCAGTCCTTGCTGAAAGACA (R). QPCR was performed with Platinum SYBR Green qPCR SuperMix-UDG mix (Invitrogen) using the Mx3000P System (Stratagene). The QPCR cycling program consisted of 40 three-step cycles of 15 s/95°C, 30 s/TA (annealing temperature), and 30 s/72°C.

Serum biomarker assessment

Serum levels of carboxy-terminal pro-peptide of collagen 1 (PICP) were quantified using an ELISA assay from Takara Biochemicals (Osaka, Japan) according to the manufacturer's instructions. Assay sensitivity was 2.0 ng/mL. Serum levels of amino-terminal pro-peptide of collagen 1 (PINP) and carboxy-terminal telo-peptide of collagen 1 (CITP) were measured using

RIA from Orion Diagnostica (Espoo, Finland). For each of these markers assay sensitivity was 13.0, 1.9, and 0.5 ng/mL, respectively.

Original blots and gels

Figure S1

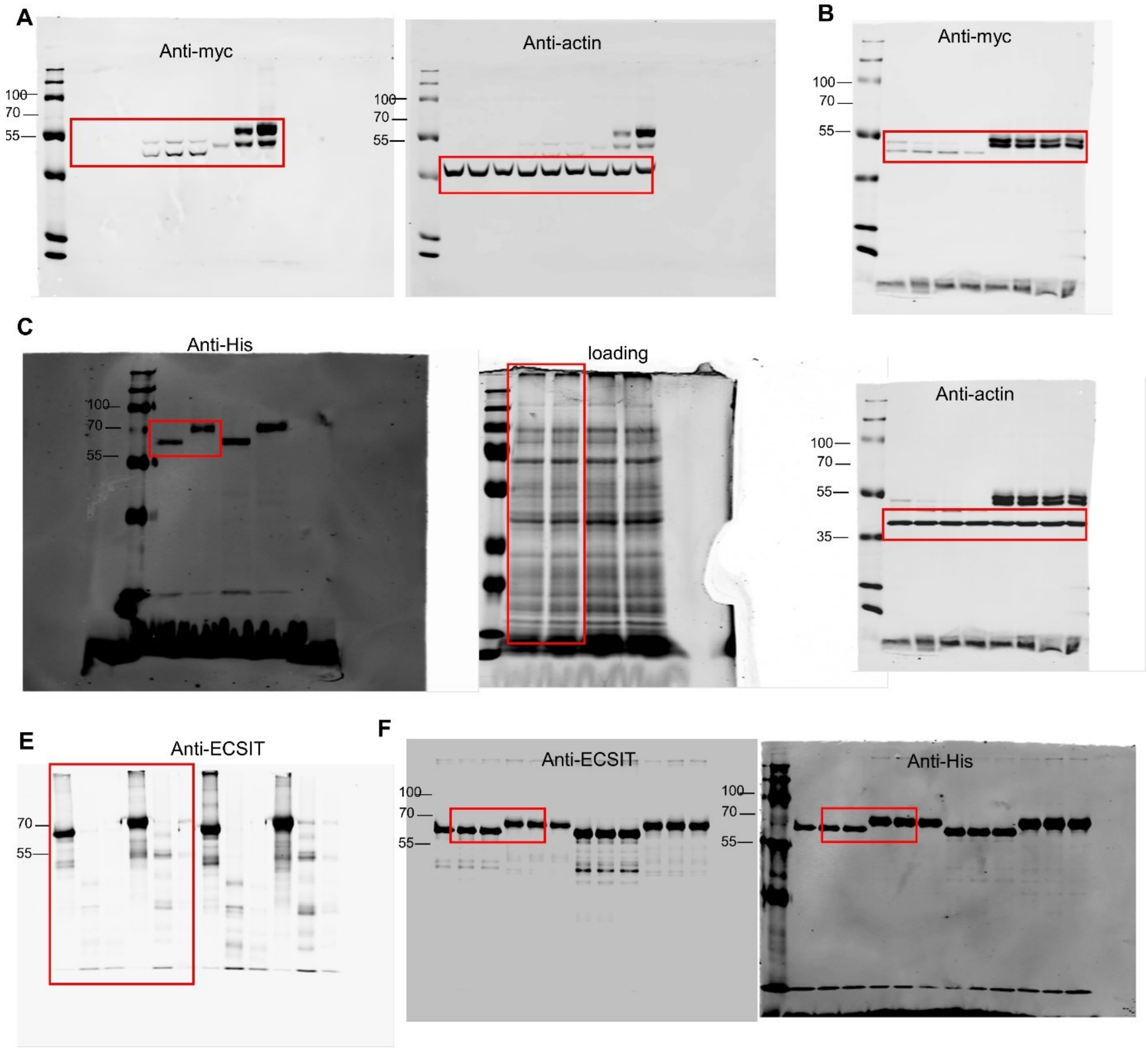

Figure 1A

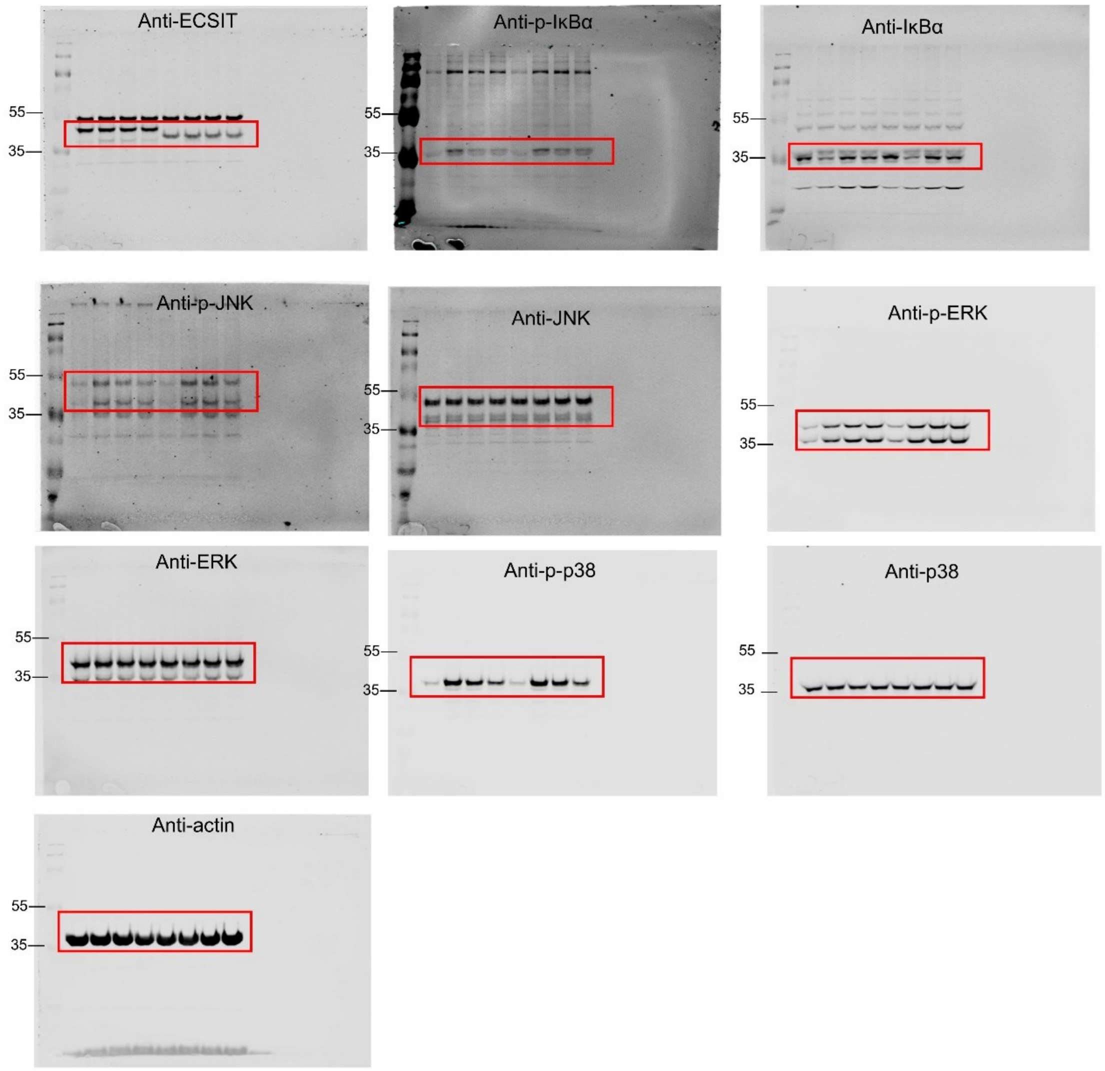

Anti-ECSIT
55
35
Anti-p-IκBα
55
35
Anti-IκBα
55
35
Anti-p-JNK
55
35
Anti-JNK
55
35
Anti-p-ERK
55
35
Anti-ERK
55
35
Anti-p-p38
55
35
Anti-p38
55
35
Anti-actin
55
35

Figure 1D

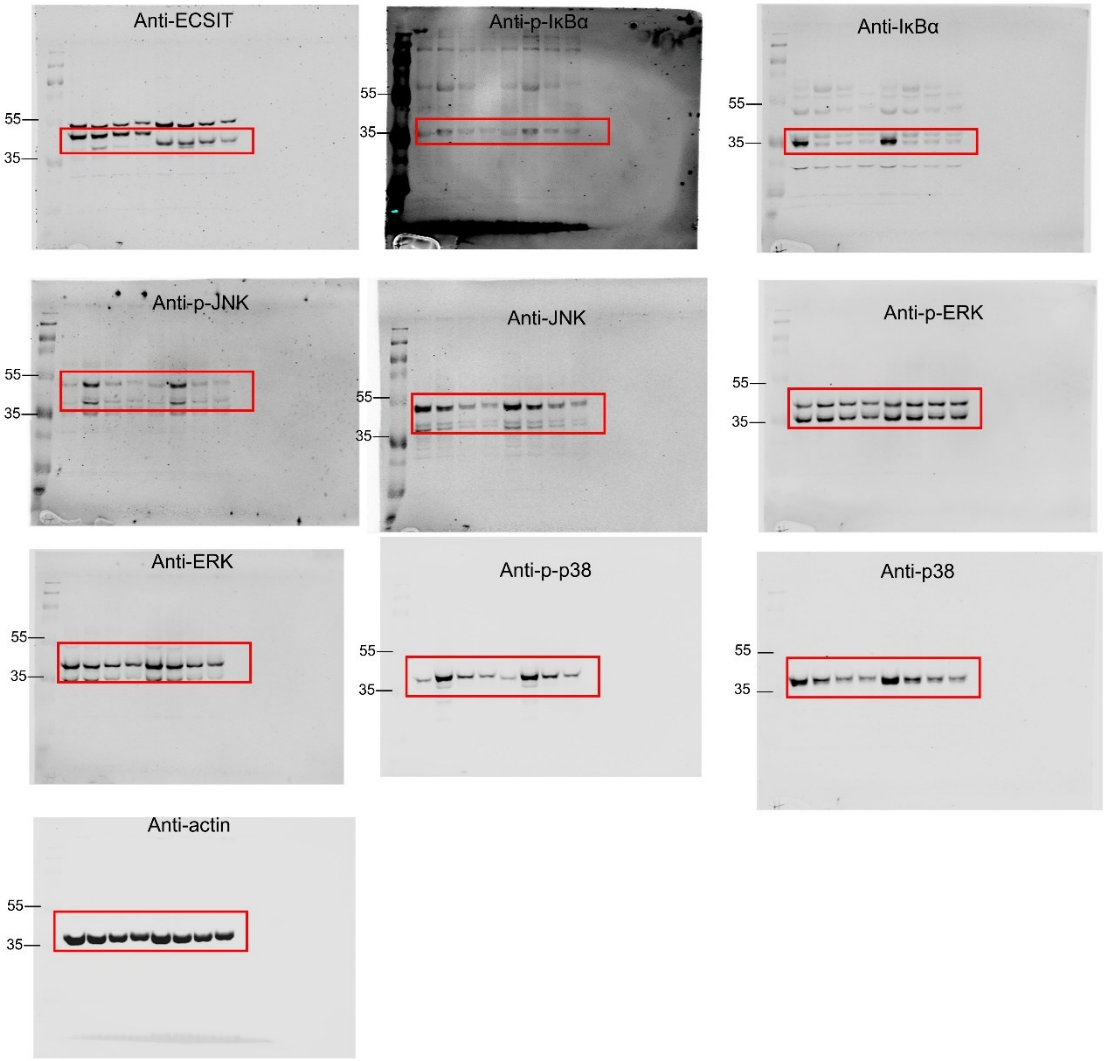
Anti-ECSIT
Anti-p-IκBα
Anti-IκBα
Anti-p-JNK
Anti-JNK
Anti-p-ERK
Anti-ERK
Anti-p-p38
Anti-p38
Anti-actin
55
35

Figure S3

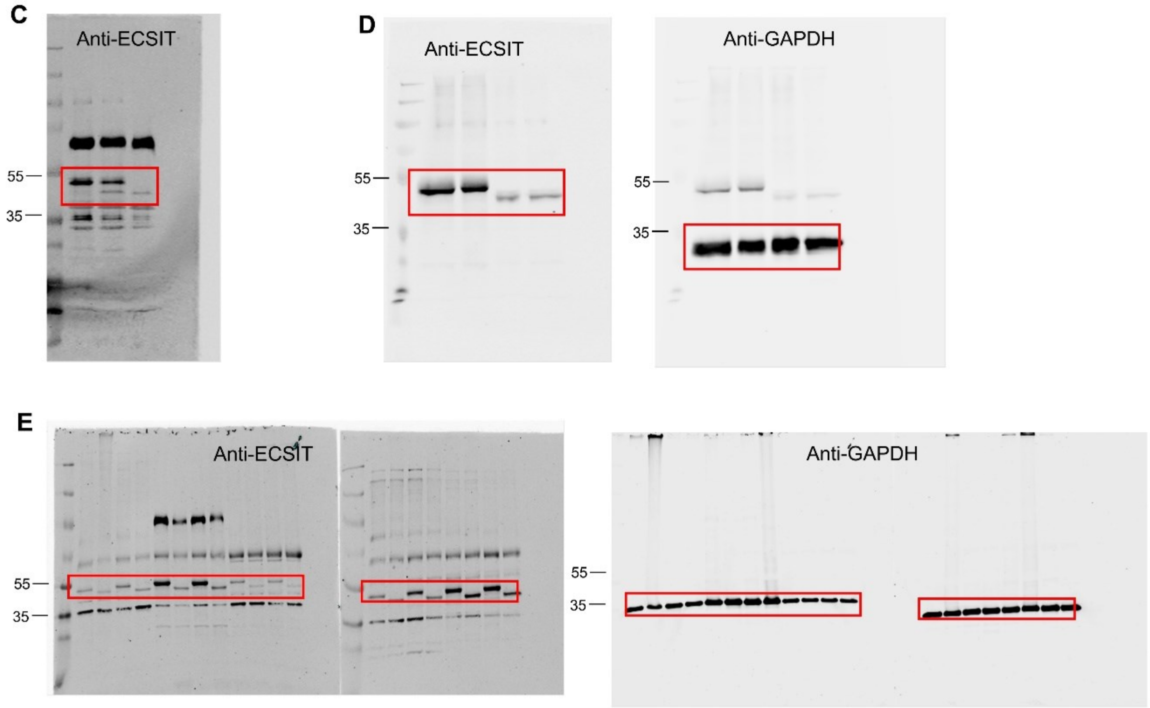

Figure S4C

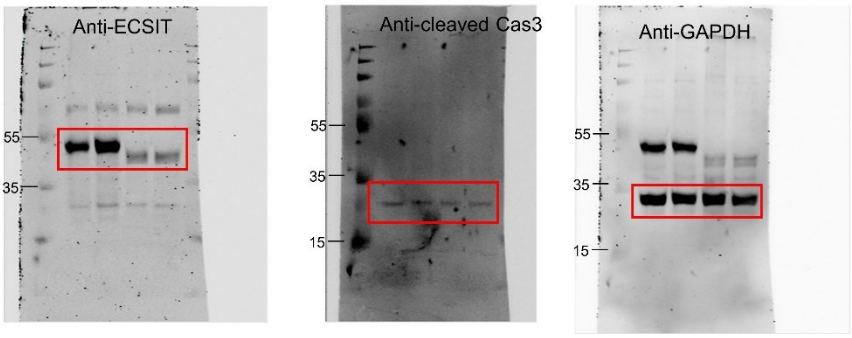

Figure 4E

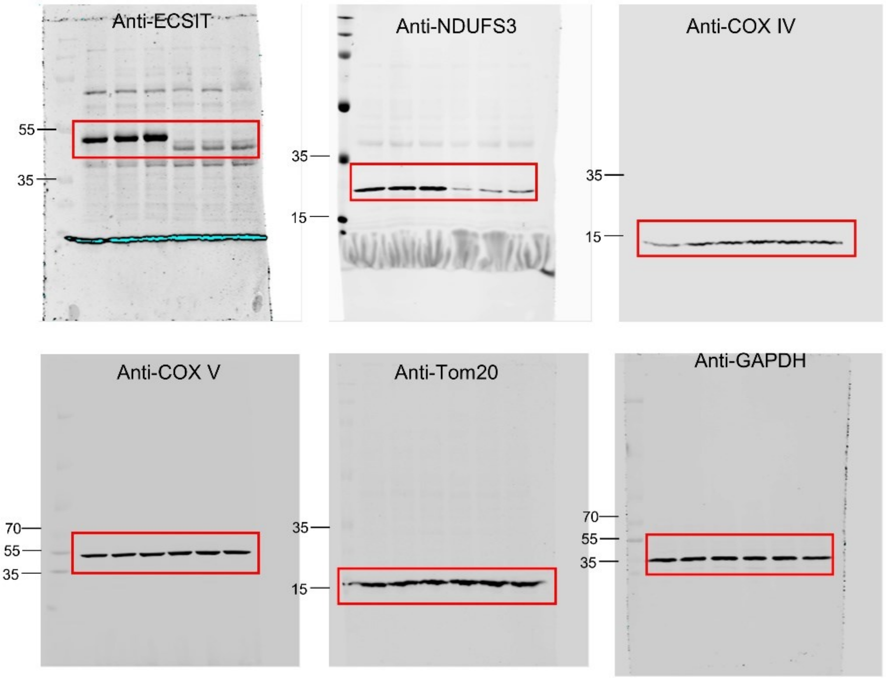

394

Figure 5C

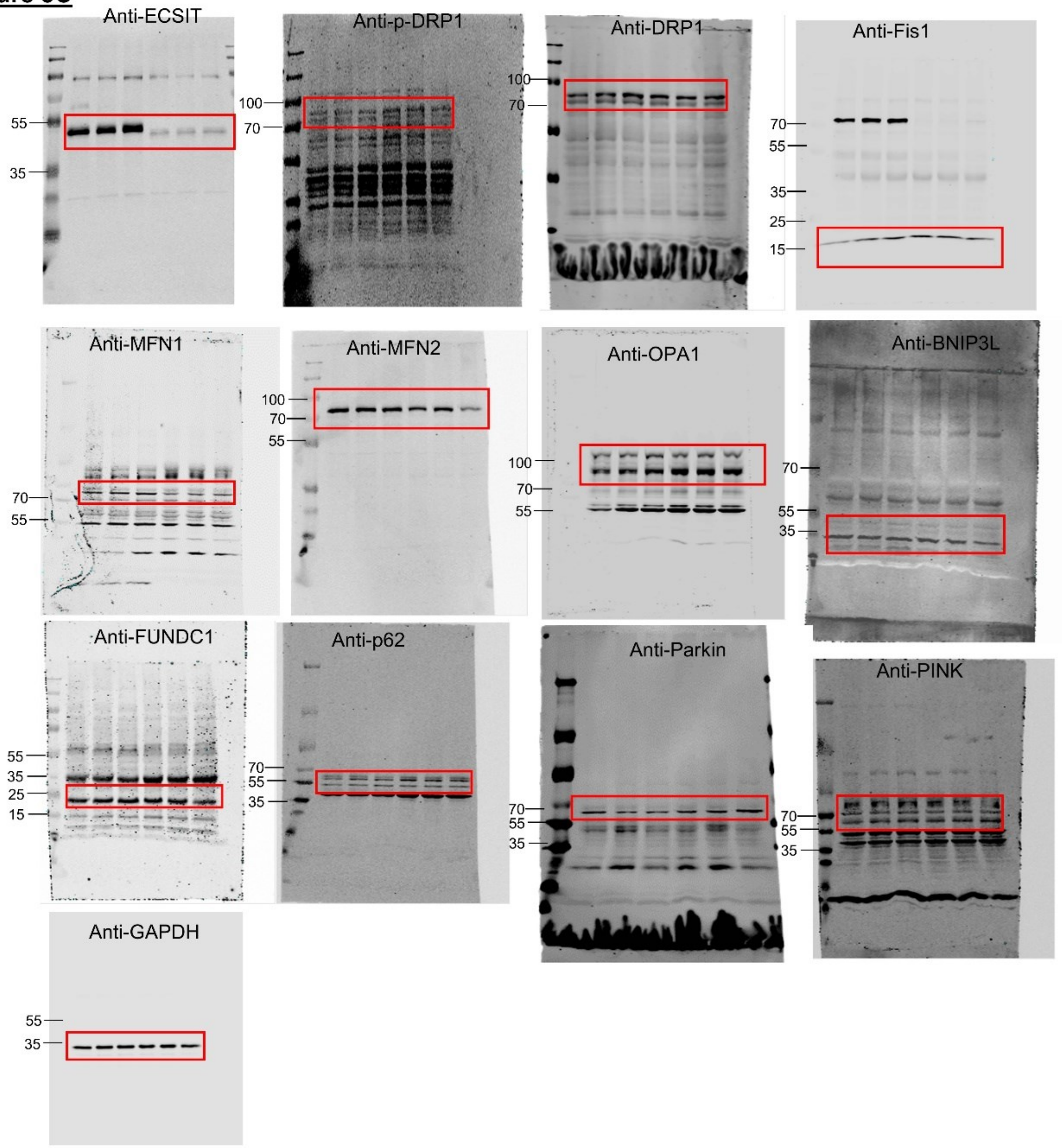
Anti-ECSIT
Anti-p-DRP1
Anti-DRP1
Anti-Fis1
Anti-MFN1
Anti-MFN2
Anti-OPA1
Anti-BNIP3L
Anti-FUNDC1
Anti-p62
Anti-Parkin
Anti-PINK
Anti-GAPDH

Figure 6A **Figure 6F**

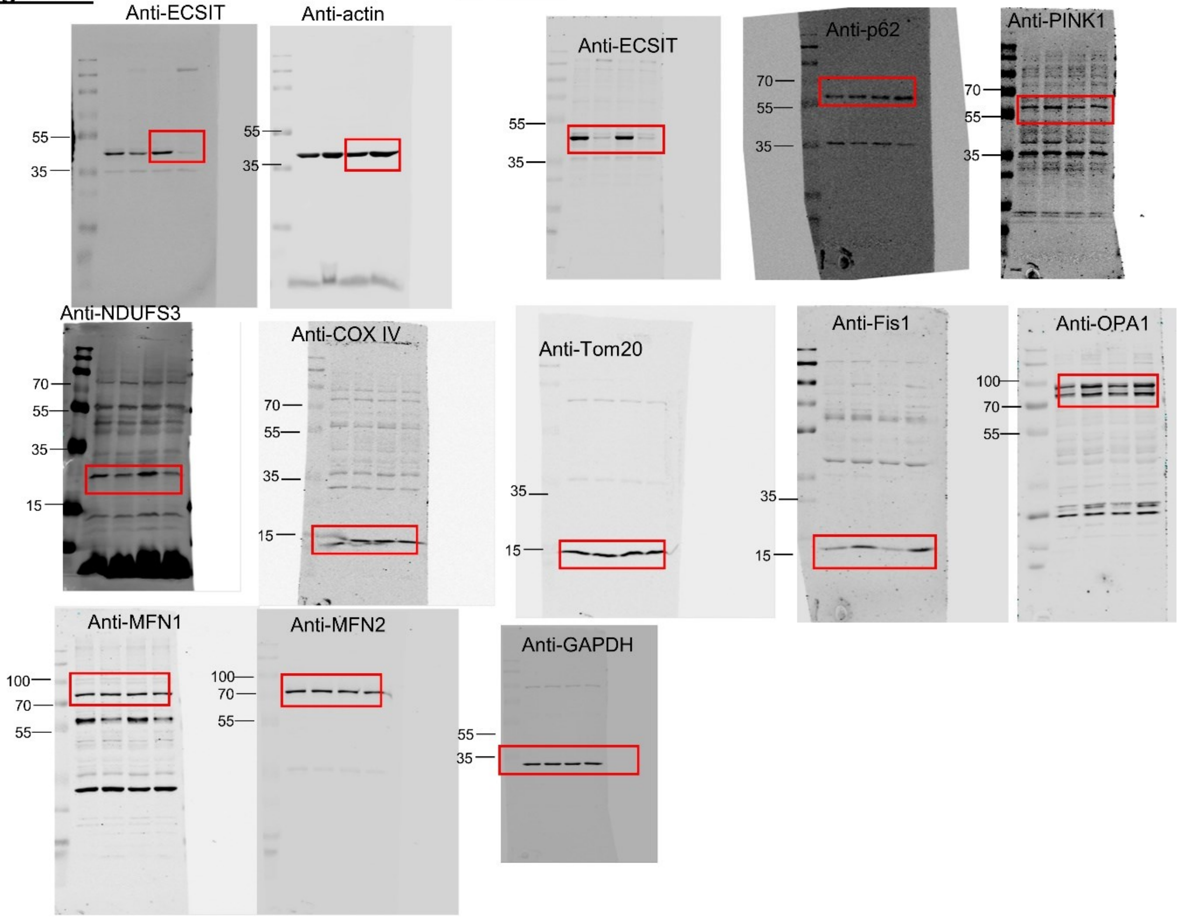

Figure 6D

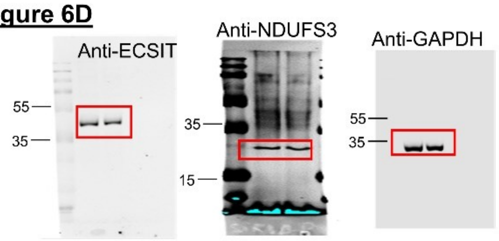

Figure S1D

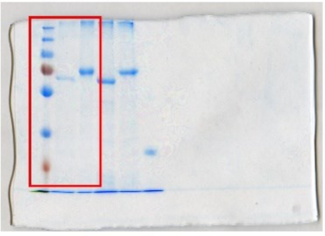

Figure 4F

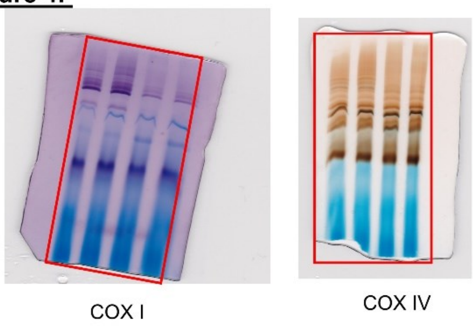

Figure 4G

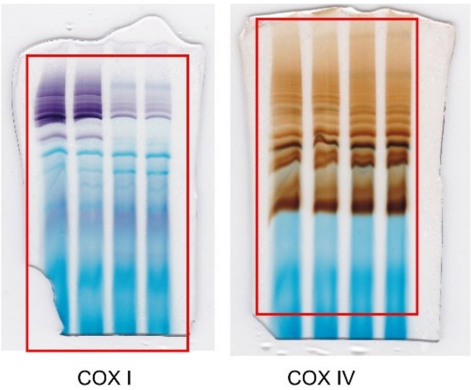

Figure 6E

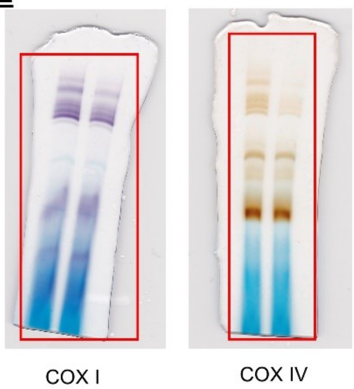

Supplement: Supplemental data [file jciinsight-6-142801-s240.pdf]
